# Profiling of humoral immune responses to norovirus in children across Europe

**DOI:** 10.1038/s41598-022-18383-6

**Published:** 2022-08-22

**Authors:** Nele Villabruna, Ray W. Izquierdo-Lara, Claudia M. E. Schapendonk, Erwin de Bruin, Felicity Chandler, Tran Thi Nhu Thao, Brenda M. Westerhuis, Janko van Beek, Louise Sigfrid, Carlo Giaquinto, Herman Goossens, Julia A. Bielicki, Malte Kohns Vasconcelos, Pieter L. A. Fraaij, Marion P. G. Koopmans, Miranda de Graaf

**Affiliations:** 1 Department of Viroscience, Erasmus MC, Wytemaweg 80, 3015 CN Rotterdam, The Netherlands; 2 Institute of Virology and Immunology (IVI), Bern, Switzerland; 3 Department of Infectious Diseases and Pathobiology, Vetsuisse Faculty, University of Bern, Bern, Switzerland; 4 Department of Microbiology, Blavatnik Institute, Harvard Medical School, Boston, MA USA; 5 Nuffield Department of Medicine, Centre for Tropical Medicine and Global Health, University of Oxford, Oxford, UK; 6 Division of Paediatric Infectious Diseases, Department of Women’s and Children’s Health, University Hospital of Padua, Padua, Italy; 7 Laboratory of Medical Microbiology, Vaccine & Infectious Disease Institute (VAXINFECTIO), University of Antwerp, Antwerp, Belgium; 8 Paediatric Infectious Disease Research Group, Institute for Infection and Immunity, St George’s University of London, London, UK; 9 Department of Infectious Diseases and Vaccinology, University of Basel Children’s Hospital (UKBB), Basel, Switzerland; 10 Institute of Medical Microbiology and Hospital Hygiene, Heinrich Heine University Düsseldorf, Düsseldorf, Germany; 11 Sophia Children’s Hospital, Erasmus MC, Rotterdam, The Netherlands

**Keywords:** Immunology, Microbiology, Virology

## Abstract

Norovirus is a leading cause of epidemic acute gastroenteritis. More than 30 genotypes circulate in humans, some are common, and others are only sporadically detected. Here, we investigated whether serology can be used to determine which genotypes infect children. We established a multiplex protein microarray with structural and non-structural norovirus antigens that allowed simultaneous antibody testing against 30 human GI and GII genotypes. Antibody responses of sera obtained from 287 children aged < 1 month to 5.5 years were profiled. Most specific IgG and IgA responses were directed against the GII.2, GII.3, GII.4, and GII.6 capsid genotypes. While we detected antibody responses against rare genotypes, we found no evidence for wide circulation. We also detected genotype-specific antibodies against the non-structural proteins p48 and p22 in sera of older children. In this study, we show the age-dependent antibody responses to a broad range of norovirus capsid and polymerase genotypes, which will aid in the development of vaccines.

## Introduction

Norovirus is the leading cause of non-bacterial gastroenteritis, resulting in an estimated 685 million infections per year and over 200,000 deaths globally^1,2^. In low-income countries, norovirus-associated deaths occur primarily among young children, whereas in high-income countries, norovirus-associated mortality is highest in adults > 55 years^3^. *Norovirus* is a genus within the *Caliciviridae* family*,* which has a single-stranded positive-sense RNA genome, that is organized into three open reading frames (ORFs). In human norovirus, ORF1 encodes for a polyprotein that is co-translationally cleaved into six non-structural (NS) proteins, including the RNA-dependent RNA polymerase (RdRp). ORF2 and ORF3 encode the major and minor capsid proteins (VP1 and VP2) that make up the viral capsid.

As noroviruses frequently undergo intergenogroup recombination, a nomenclature system was adopted that independently assesses genotypes of ORF1 and ORF2/3. Based on the amino acid homology of the VP1, noroviruses are categorized into ten genogroups (GI–GX) that are further subdivided into at least 48 capsid genotypes and, based on the RdRp sequence, into at least 60 polymerase genotypes (P-types)^4^. The majority of infections are caused by GI and GII viruses, of which GII.4 noroviruses are the most predominant^4–6^. In addition, noroviruses have also been found in a broad range of animal species, raising the question of whether animal genotypes could enter the human population, especially animal noroviruses that cluster with viruses in genogroups found in humans: such as genotypes GII.11/18/19 (detected in pigs) and GIV.2 (detected in dogs and cats)^4^.

Estimating the prevalence of norovirus infections and that of different norovirus genotypes, in particular, is challenging. Unless they are part of a large outbreak or show severe symptoms, most people will likely not seek medical treatment^7^. In addition, depending on the setting, region, age, and possibly genotype, 7% to 30% of individuals are presumed to be infected without developing symptoms^8^. As an alternative approach to pathogen detection in stool samples, serology is commonly used to estimate the prevalence in a population. Serological studies representing different populations globally have identified a prevalence of antibodies to noroviruses ranging between 80 and 100% in adults^9–24^. Most serology studies used virus-like particles (VLPs) of GI.1, GII.3, and GII.4^9,17,25,26^, and only a few have investigated antibody responses to other norovirus genotypes^15,27,28^. These VLPs consist of 90 major capsid protein (VP1) dimers and are antigenically comparable to native virions^29^. The VP1 consists of the C-terminal protruding (P) domain, which contains the major antigenic epitopes, and the conserved N-terminal shell (S) domain. Cross-reactive antibodies against the conserved S domain make it challenging to measure genotype-specific antibody responses^30^. This is further complicated by the fact a patient’s infection history impacts the magnitude and depth of the antibody response elicited in later infections^31–33^.

To understand the exposure to different genotypes, one can study cohorts of sera obtained from children with more specific antibody assays. In contrast to adults, young children have had less frequent exposures; therefore, serum samples from children are more likely to come from individuals with a more specific reaction.

To improve the detection of genotype-specific antibodies, P particles consisting of the variable P domain have been used as an alternative to VLPs^34,35^. We have previously developed a multiplex protein microarray using P particles which allows high throughput testing for genotype and genogroup-specific antibodies^28^. In addition, NS proteins can be used as antigens. In mice, immunization with the ORF1 polyprotein resulted in some protection from murine norovirus infection, which prompted speculation about the existence of protective antibodies that target non-capsid proteins^36^. Antibodies against GI.1 protease and p22, as well as a polyprotein (VPg-Protease-RdRp), have been reported^37–39^. In contrast to the more conserved C-terminal proteins, the N-terminal proteins (p48, NTPase, and p22) are more diverse than other parts of the genome. This raises the question of whether exposure to polymerase genotypes could be assessed independently, thereby allowing serosurveys for exposure to recombinant viruses^40^.

In this study, we set up a protein microarray that we validated using pre- and post-infection sera from 120 children. To investigate the diversity of norovirus exposure in European children, we studied the age-related seroprevalence of antibodies against 30 P particles representing the human norovirus diversity with sera collected from 287 children aged < 1 month to 5.5 years to understand the acquisition of genotype and genogroup-specific antibodies with increasing age. We furthermore investigated the potential of NS antigens to be used to assess ORF1 circulation by measuring their antigenicity and seroprevalence.

## Results

### IgG and IgA response pre- and post-norovirus infection

To validate that IgG and/or IgA antibody responses against the P particles are elicited by norovirus infection, we tested four pre- and post-infection sera of immunocompetent adults who had a norovirus infection with a known capsid genotype: either GI.1, GII.3, or GII.4 (New Orleans 2009 and Den Haag 2006b). The polymerase genotypes were unknown. Sera were tested against P particles of nine GI and 18 GII human noroviruses, as well as three porcine genotypes (GII.11/18/19) that are closely related to human norovirus. Three of four sera showed the highest increase in antibody titer against the P domain of the homologous genotype, against which post-infection titers were increased by 63-fold (GII.4 2006b), 1.2-fold (GII.3), and 5-fold (GII.4 2009) (Table 1). The GI.1 sera showed post-infection titers of > 2560 against GI.1, GI.3, and GI.7. All four sera had pre-existing titers (> 40) against the majority of genotypes, and an increase in antibody titer post-infection was also observed against heterologous genotypes. This was most evident for the GI.1 and the GII.4 2006b serum that had > 4-fold increased post-infection titers against several GI and GII genotypes (Table 1, shown in red). Cross-reactive responses were primarily directed against genotypes belonging to the same genogroup.

**Table 1:**
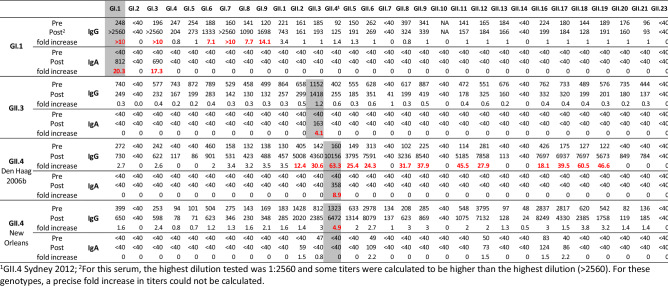
IgG and IgA titers against P particles in pre- and post-infection sera.

Marked in red are the titers that were ≥ 4-fold increased in post-infection sera compared to pre-infection sera.

Shaded in grey are the antibody responses against the homologous infecting genotypes. Antibody responses against the non-structural proteins are listed in supplementary table 1.

The IgA response was lower and more specific. The GII.3 and GII.4 2006b had specific responses against the homologous genotypes (Table 1). The GI.1 serum showed an increase in IgA titers against GI.1 and GI.3, similar to the IgG response. The pre-infection serum of the GII.4 2009 infected individual was the only serum with pre-existing IgA antibody titers (≤ 83) and no detectable IgA response against the homologous genotype.

### Profiling of the IgG immune responses against norovirus genotypes in children

To study genotype-specific seroprevalence in children, we tested a cohort of 287 children’s sera. The cohort included sera of children aged < 1 month to 5.5 years and was collected between 2016 and 2019 from 16 sites in seven European countries and is further described in Kohns Vasconcelos et al*.*^41^. Sera were tested on the protein array, and titers above 40 were considered positive for an antigen.

When dividing the sera into four age groups (see “Materials and methods” section), an age-related increase in titers against most genotypes was detected. Overall, norovirus seroprevalence was highest for children in the 0–6 months group, in which 95.3% of sera recognized at least one genotype (Supplementary Fig. 1a). Seroprevalence decreased to 59.6% (> 6–12 months) and increased to 78.5% (> 1–1.5 years) and 84.7% (> 1.5–5.5 years). This high seroprevalence remained when the cut-off for titers, considered positive, was increased from > 40 to > 100. When using the higher cut-off, the overall seroprevalence decreased by 3.5% to 10.6% in all groups except in the > 6–12 months group, where it decreased by 23.1% (59.6% to 36.5%), indicative of a high number of sera with low titers (Supplementary Fig. 1a).

**Figure 1:**
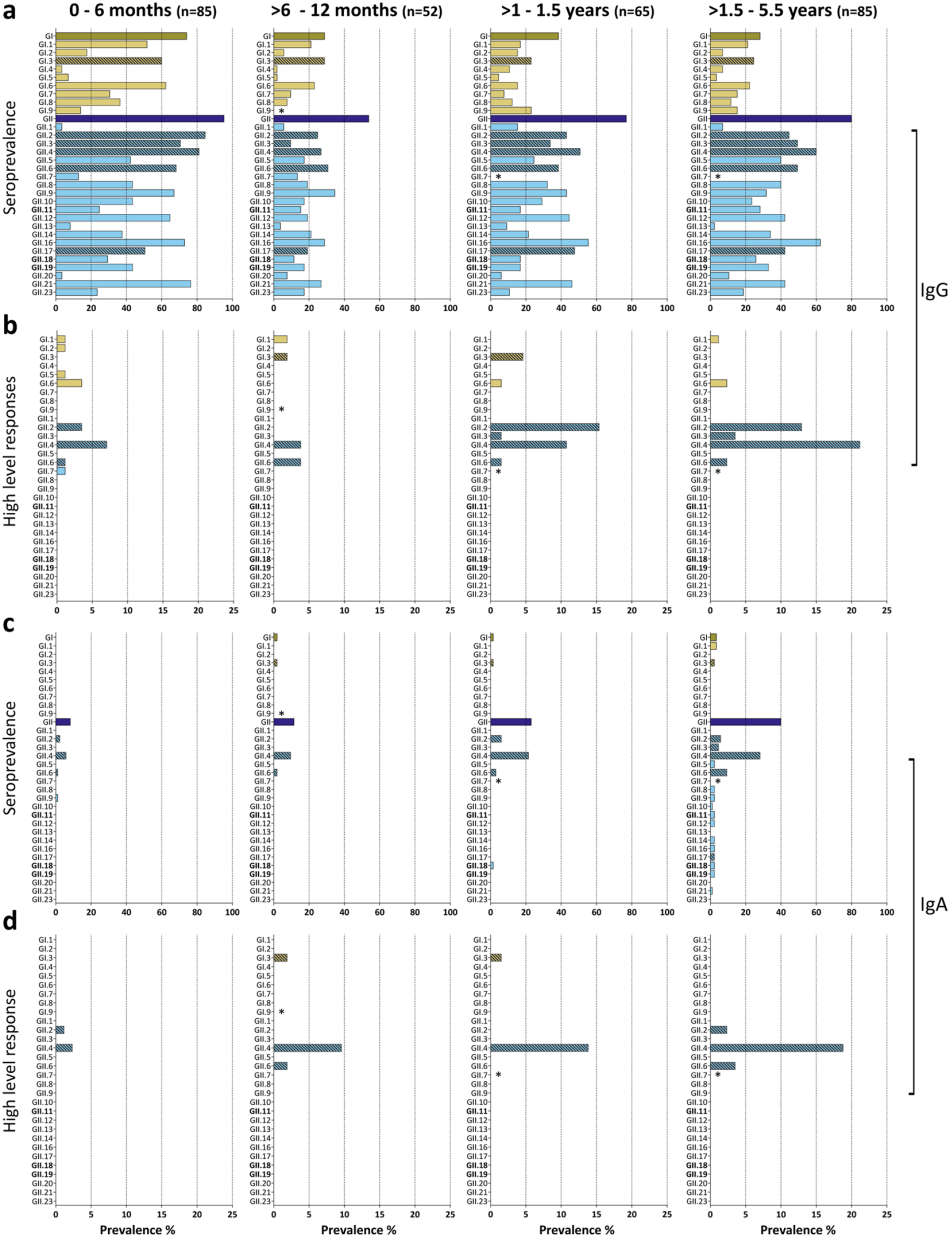
Genotype and genogroup-specific IgG and IgA responses. **(a)** IgG and **(c)** IgA seroprevalence in four age groups against norovirus P particles representing the human norovirus diversity. The total seroprevalence for GI and GII is also shown (dark bars). Sera with an antibody titer > 40 were considered positive. **(b)** IgG and **(d)** IgA immunodominant response sera were defined as having an antibody response against one P particle ≥ 4-fold higher than against the other genotypes. The genotypes most commonly reported in NoroNet during outbreaks are marked with a pattern. *Marks antigens that were not spotted. In bold are the porcine genotypes.

**Figure 2:**
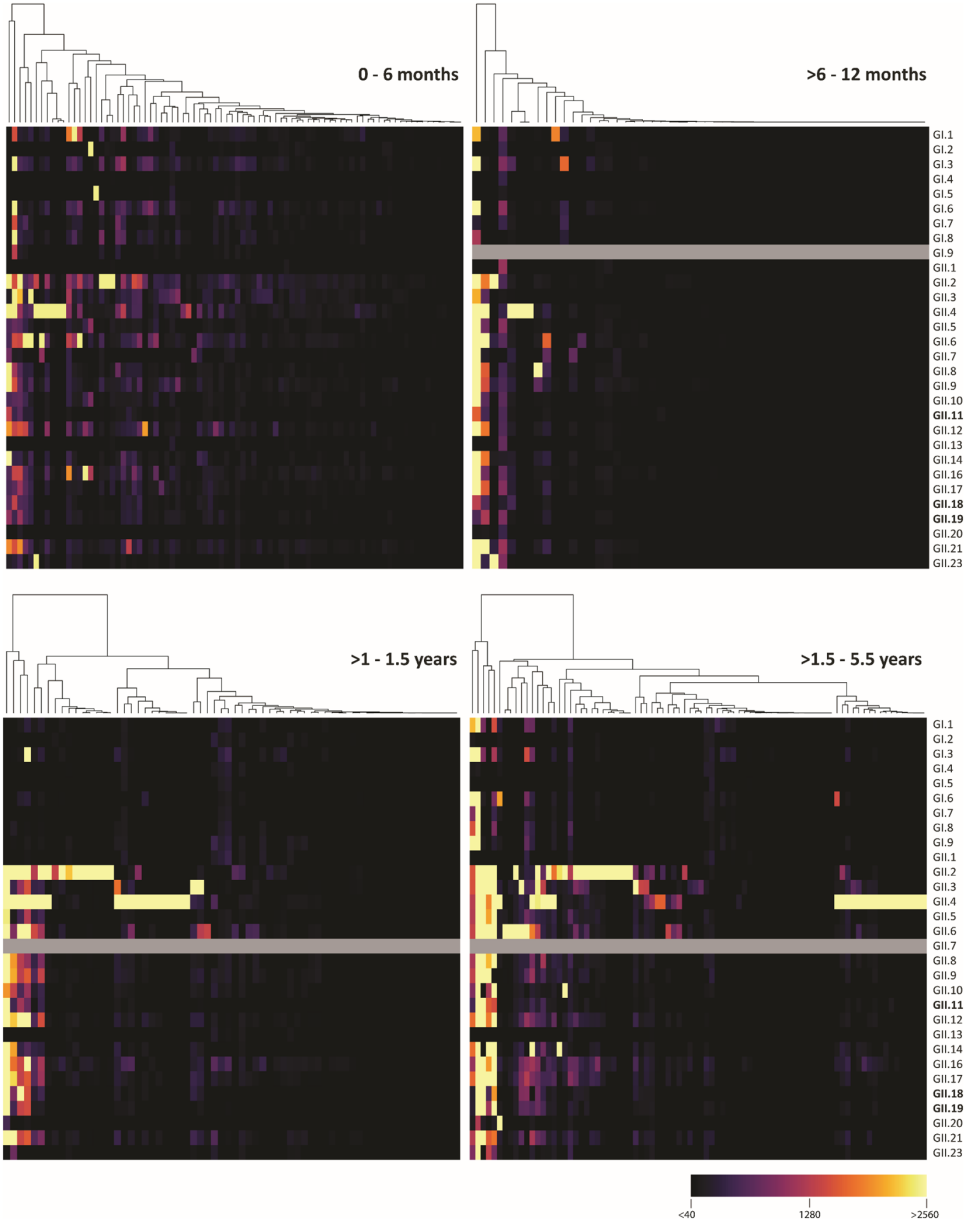
IgG responses against norovirus. Heat map of sera titers against P particles in the four age groups. Each column represents the antibody titers of one serum. Yellow depicts titers > 2560 and black titers < 40. Responses were clustered by antibody response. Grey marks antigens that were not spotted. In bold are the porcine genotypes.

When seroprevalence against each genotype was analyzed, seroprevalence was highest in the 0–6 months-old children, in whom seroprevalence was > 80% for some genotypes, representing maternal immunity (Figs. 1a, 2). In children > 6–12 months, seroprevalence decreased to < 20% for most genotypes. Only GI.3, GII.2, GII.4, GII.6, GII.9, GII.16, and GII.21 reached 20–40%. These levels increased in the older age groups and the highest increase in seroprevalence was seen for GII.4, GII.3, GII.6, and GII.16, with a seroprevalence of 50–62% (> 1.5–5.5 years). Antibodies were also detected against porcine noroviruses, for which the seroprevalence increased up to 28–33% (> 1.5–5.5 years).

Interestingly, seroprevalence against GI genotypes was high (74.1%) in the 0–6 months group and decreased to 28.8% in the > 6–12 months group, but in contrast to seroprevalence against GII, which increased again in older age groups, overall GI seroprevalence remained < 40%.

Few sera had an IgG response against a single genotype, and some responses were likely due to cross-reactivity. To exclude potentially cross-reactive sera, we selected sera with an immunodominant antibody response, as evidenced by antibody titers against one genotype that was ≥ 4-fold higher than other genotypes (Fig. 1b). Assuming limited cross-reactivity between genogroups, analyses were done separately for GI and GII responses^28,42–44^. Immunodominant responses were detected in 20.0% (0–6 months), 11.5% (> 6–12 months), 35.4% (> 1–1.5 years), and 43.5% (> 1.5–5.5 years) of the sera. Immunodominant antibody responses to GI genotypes (GI.1, GI.2, GI.3, GI.5, and GI.6) and GII.6 were detected in all groups and did not increase with age. In contrast, immunodominant antibody responses were directed to GII.2, GII.3, and GII.4 increased with age (Fig. 1b), suggesting more GII infections in the two older age groups. No immunodominant responses towards rare genotypes were found, but we detected a few sera with high titers against a limited number of antigens that included rarely detected genotypes (i.e., GII.8 and GII.23) (Fig. 2).

These immunodominant responses, except for GII.17, agree with the NoroNet norovirus surveillance data from Europe (Supplementary Fig. 2a), in which 48.4% of reported outbreaks are linked to GII.4, followed by GII.2 (11.7%), GII.17 (9.6%), GII.3 (6.5%), and GII.6 (5.3%). Of the GI genotypes, GI.3 (3.7%) is most commonly found.

In conclusion, we could detect antibody responses against all evaluated genotypes, of which some responses were likely due to cross-reactivity. However, immunodominant responses were limited to a few genotypes that are also most commonly reported in outbreak surveillance.

### Profiling of the IgA immune responses against norovirus genotypes in children

The age-related pattern was different for the IgA response, apparent by the low IgA seroprevalence in the youngest groups, 8.2% (0–6 months) and 11.5% (> 6–12 months). IgA titers increased with age to 23.1% (> 1–1.5 years) and 41.2% (> 1.5–5.5 years) (Supplementary Fig. 1b). When the cut-off for antibody titers was increased from > 40 to > 100, the seroprevalence decreased by ≤ 1.6% in all groups except for the > 1.5–5.5 years group, in which it decreased from 41 to 33% (Supplementary Fig. 1b).

The IgA seroprevalence was limited to a few genotypes: GI.1, GI.3, GII.2, GII.3, GII.4, and GII.6, and most responses were detected against GII.4 (Fig. 1c). Except for two sera in the oldest age group that reacted against all GII genotypes, the majority of IgA responses were limited to one or two genotypes, suggesting lower levels of cross-reactivity compared to IgG, shorter persistence, or lower overall IgA levels. Most immunodominant responses were detected against GII.4, and this number increased in older children (Fig. 1d). Genotype-specific responses were rare in the 0–6 months group (3.5%), while 13.5% (> 6–12 months), 15.4% (> 1–1.5 years), and 24.7% (> 1.5–5.5 years) showed immunodominant responses. As for IgG, immunodominant responses against GI did not increase with age. The predominantly anti-GII.4 IgA responses were associated with a high anti-GII.4 IgG response (Spearman’s rank correlation coefficient, r = 0.6323, p < 0.0001). All the sera with anti-GII.4 IgA titers > 40 had anti-GII.4 IgG titers > 1000 and 89.6% had anti-GII.4 IgG titers > 2560.

In conclusion, norovirus-specific IgA seroprevalence increased with age in children, and the response was predominantly directed against GII.2 and GII.4, the most common genotypes reported in NoroNet.

### Antigenicity of non-structural norovirus proteins

**Figure 3:**
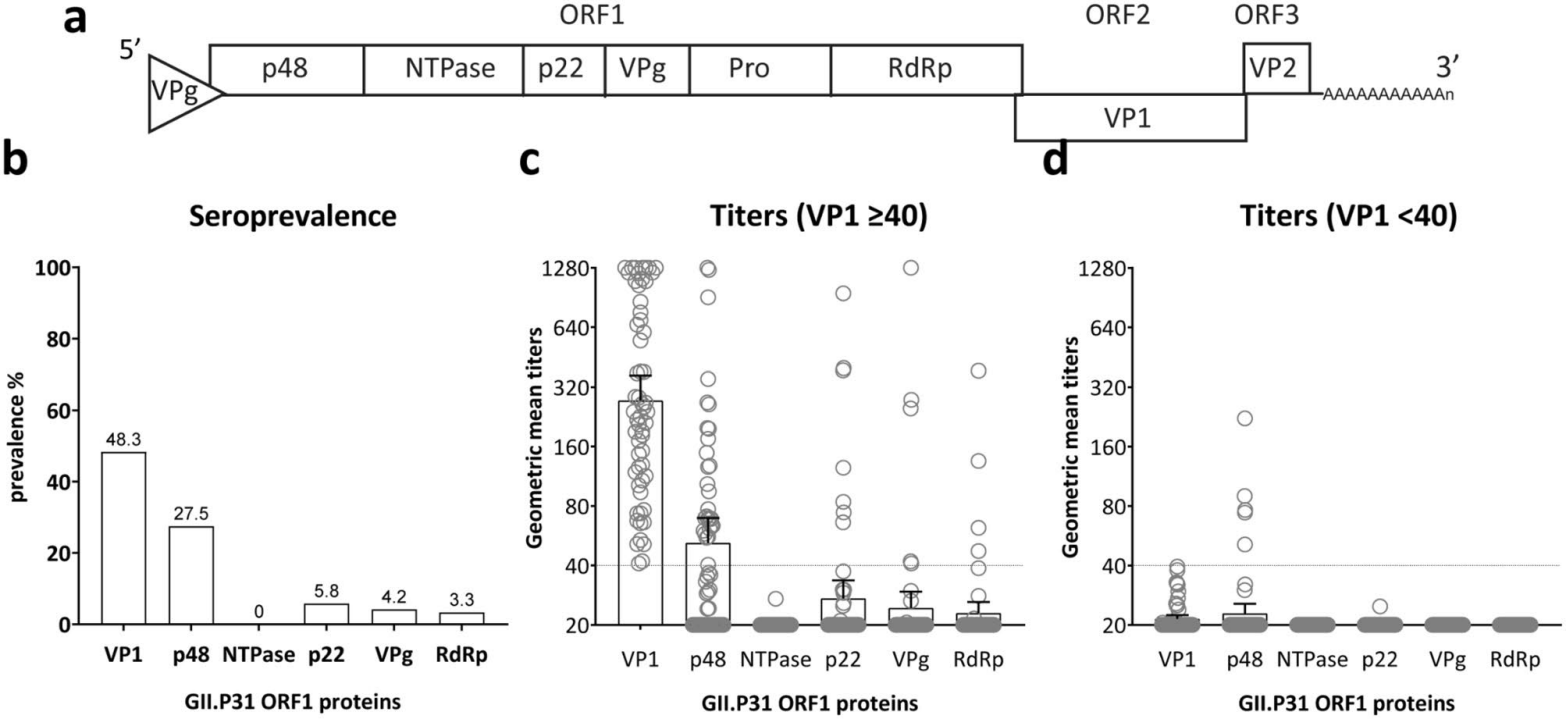
Antibody responses against the non-structural (NS) proteins from GII.4 Sydney[P31]. Sera of 120 children were tested for antibody response against norovirus antigens on a protein microarray. **(a)** Organization of the three open reading frames (ORF1-3) of the human norovirus genome. Six NS proteins are encoded by ORF1, whereas VP1 and VP2 are encoded by ORF2 and ORF3, respectively. **(b)** Seroprevalence (antibody titers > 40) against the capsid (VP1) and the NS proteins. The percentage of sera with antibody titers > 40 is noted on top of the bars. Geometric mean and 95% confidence interval are shown for sera with VP1 antibody titers **(c)** > 40 and **(d)** < 40. Negative sera were set to a titer of 20.

Due to recombination between ORF1 and ORF2, some ORF1s are associated with several capsid types. Therefore, antibodies against the VP1 give an indication of the circulating ORF2 but not necessarily about the respective ORF1. Antigenicity is not well described for the NS proteins. To select suitable antigens for the protein microarray, we first tested the IgG seroprevalence against the ORF1 proteins and the capsid of a global dominant GII.4 Sydney[P31] strain. A historic cohort, including sera of 120 children, was used to determine the antigenicity and the seroprevalence of the NS proteins. The cohort included Dutch children between nine months and two years of age, from which sera were collected between 2010 and 2015. Seroprevalence was highest against the VP1 antigen, for which 48.3% of the sera had titers > 40 (Fig. 3a,b). Of the NS proteins, antibodies binding to the p48 antigen were detected in 27.5% of the sera. Antibodies against the other NS proteins were detected in 5.8% (p22), 4.2% (VPg), and 3.3% (RdRp). The NTPase was the only antigen to which no antibody binding was observed (the protease failed to be printed). Antibodies against NS proteins were associated with high VP1 titers (Fig. 3c,d); only five of the sera with p48 titers > 40 had no antibodies against VP1.

In conclusion, IgG responses against p48, p22, VPg, and RdRp were detected in serum samples from children, with the highest seroprevalence against p48 followed by p22, which were further used as antigens on the array to increase the odds of capturing genotype-specific antibodies.

### Profiling of IgG and IgA immune responses against norovirus polymerase genotypes in children

To assess the diversity of ORF1 circulation in children, we tested antibody responses of a subset of 247/287 sera against p48 and p22 of the four most commonly detected GI.P genotypes (GI.P2, GI.P3, GI.P4, GI.P11), eight human GII.P genotypes (GII.P2, GII.P4, GII.P7, GII.P16, GII.P17, GII.P21, GII.P31, GII.PNA2), and porcine GII.P18. For p48, additional GIV.P1 and animal noroviruses were included: GIII.P2 (cow), GVI.P1 (cat), GVII.P1 (dog), and GX.P1 (bat). Table 2 lists the ORF2 sequences that are most commonly reported in combination with our selection of ORF1 sequences.

**Table 2:** The most common ORF1-ORF2 combinations, taken from Ref.^45^.

ORF1	ORF2
GI.P2	GI.2
GI.P3	GI.3
GI.P4	GI.4
GI.P11^1^	GI.6
GII.P2	GII.2
GII.P4	GII.4
GII.P7^2^	GII.6
GII.P16	GII.2/GII.4
GII.P17	GII.17
GII.P18	GII.18
GII.P21	GII.3
GII.P31	GII.4
GII.PNA2^3^	GII.NA2^3^
GIII.P2^2^	GIII.2
GIV.P1^2^	GIV.1
GVI.P1^2^	GIV.2
GVII.P1^2^	GVII.1
GX.P1^2^	GX.1

^1^Formerly GI.Pb.

^2^Only p48 was expressed.

^3^Closest to GII.24[P24].

The age-related pattern was similar to the IgG response against the P particles; 42.9% in the 0–6 months group recognized at least one antigen, followed by a drop to 23.8% (> 6–12 months) and an increase in older children, 61.8% (> 1–1.5 years) and 65.0% (> 1.5–5.5 years) (Supplementary Fig. 1c). When the positive cut-off titer for a sample was increased to > 100, the seroprevalence decreased in all groups by 9.5% to 12.9% (Supplementary Fig. 1c). This indicates that sera with low titers were found in all age groups.

**Figure 4:**
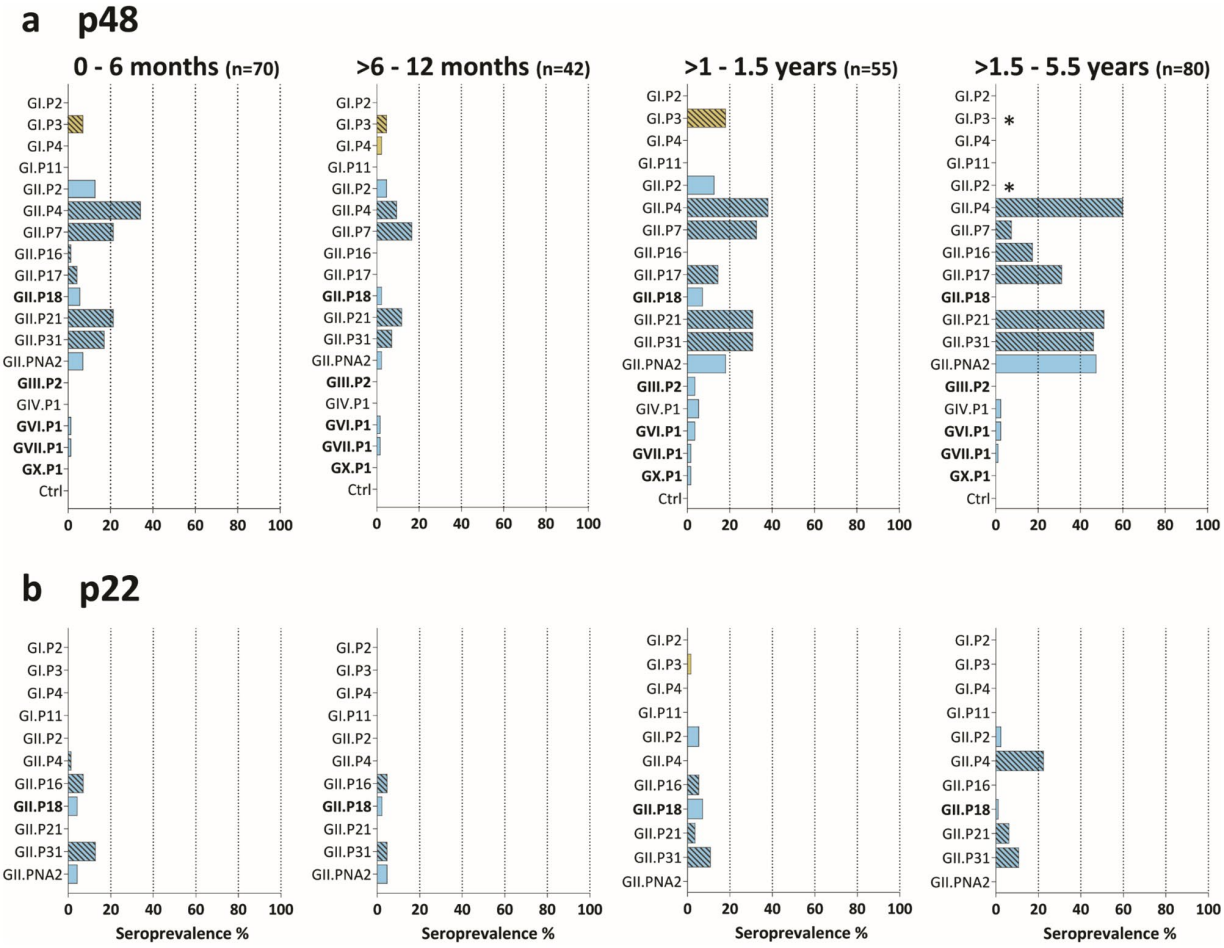
IgG response against norovirus NS proteins p48 and p22. Seroprevalence is shown for four age groups against **(a)** p48 and **(b)** p22 antigens. Sera with an antibody titer > 40 were considered to be positive. *Marks antigens that were not spotted. In bold are the animal genotypes.

The p48 seroprevalence was highest against GII.P4, GII.P31, GII.P7, and GII.P21 (Fig. 4a). GII.P4 and GII.P31 are most commonly associated with GII.4, GII.P7 with GII.6, and GII.P21 with GII.3 (Table 2). This is in agreement with the antibody responses against the P particles and surveillance data from NoroNet (Supplementary Fig. 2b). Of the GI genotypes, GI.3 was the only GI antigen recognized. In the oldest children, we detected a > 40% seroprevalence against p48 of GII.PNA2, which has only been reported once from Peru but has not been recognized as a new genotype yet. It is most closely related to GII.P24 and shares 58–69% aa similarity with the p48 of the other GIIs included on the array.

Several animal polymerase genotypes were included, and while only a few sera recognized p48 of the GIII.P2, GVI.P1, GVII.P1, and GX.P1 p48, seroprevalence against porcine GII.P18 p48 was 5.7% (0–6 months), 2.4% (> 6–12 months), and 7.3% (> 1–1.5 years). p48 of GII.P18 shares 56–63% aa identity with the p48 of the other GIIs. Antibodies against p22 were less frequently detected and restricted to GII genotypes, mostly against GII.P4 and GII.P31 (Fig. 4b).

Fifty percent (126/247) of the sera had antibody titers > 40 against at least one NS antigen. Of those, 76.8% of sera contained antibodies that recognized antigens from more than one genotype. When the VP1 responses of these sera were analyzed, 82.7% recognized > 1 VP1 genotype, and 69.7% recognized > 4. This suggests that the NS responses are increased in children with multiple exposures. This is further supported by the higher average age of children with anti-NS antibodies compared to those without (median age 16.3 months versus 9.5 months, respectively, p = 0.0006). No IgA responses against the NS proteins were detected.

In conclusion, p48 and p22 IgG responses were predominantly found against GII.P4 and GII.P31, which are highly prevalent genotypes. In general, p48-specific antibodies were more frequently detected than p22, but the responses against p48 from GII.PNA2 and GII.P18 suggest the presence of cross-reactive epitopes. Antibody responses against NS proteins were more often detected in sera with high IgG titers against several capsid genotypes, likely due to multiple infections.

## Discussion

Norovirus is a very diverse genus of viruses, with a few genotypes causing the majority of reported infections, raising questions about reservoirs and levels of undetected circulation. We hypothesized that some genotypes that are less commonly reported in NoroNet could circulate more widely in children, causing either sporadic or asymptomatic infections that are missed by surveillance and that seed outbreaks in the community.

In our study, using a panel of antigens representing a wide diversity of norovirus genotypes, we could not detect serological evidence of rare genotypes circulating widely in children. Instead, our data agree with surveillance data and indicate that norovirus circulation in children is dominated by a few genotypes. Our overall finding of an age-dependent IgG seroprevalence is in concordance with the main findings from seroepidemiological studies in children that report high seroprevalence in the first four to six months, attributable to maternal antibodies, followed by a decrease in titers and continuous low seroprevalence up to around 12 months when titers rise again^20,21,24–26,46^. The waning of maternal IgG antibodies is correlated with a peak in norovirus infection at 6 to 12 months^47–51^. The pattern for IgA was different. IgA was almost absent in sera of the youngest age group, and high titers were predominantly found in older children. This is in line with the fact that maternal immunoglobulin transferred across the placenta is IgG-dominant^52^. The IgA responses in the youngest age group show that infections do occur in these young children. The lower number of sera with IgA responses can be explained by a faster waning of the IgA antibodies. Two challenge studies with GI.1 found that IgA levels decreased after 35 days post-infection, while IgG titers remained at their peak^33,53^, although low levels of IgA levels have been reported to persist until 6months after infection^54^. The high IgA responses measured in this study were likely from a recent infection.

While most sera IgG reacted against multiple genotypes, the immunodominant antibody responses were limited to a few, namely GII.4, GII.2, GII.3, and GII.6. These were also the genotypes most commonly detected by IgA. This is in concordance with data obtained through the European norovirus surveillance network NoroNet as well as a global meta-analysis, showing that the majority of norovirus outbreaks and sporadic cases are caused by GII.4[P31], GII.4[P4], GII.2[P16], GII.3[P21], GII.6[P7] and GII.17[P17]^47,49,55–57^. Of these, GII.17 was the only genotype against which we did not detect specific responses. This might be explained by an older age of the GII.17 susceptible population, as suggested by a study in which GII.17 was only detected in adults but not children^58^.

One possibility of why we did not detect immunodominant responses against less prevalent norovirus genotypes in young children is that some genotypes might cause primarily asymptomatic infections, resulting in lower IgG and IgA responses that would be missed in serological studies. A recent challenge study showed that symptomatic infections induced a ≥ 4-fold increase in IgG and IgA titers. In contrast, asymptomatic infections were correlated with a lower viral load and significantly lower or no IgG and IgA titers^53^. GII.4 and GII.3 have been associated with a higher risk of severe gastroenteritis compared to other genotypes^49^, possibly due to the higher prevalence of GII.4 in children ≤ 1 year^59^. But whether some genotypes are more associated with asymptomatic infections is not known. However, a high norovirus diversity has been reported in asymptomatic^60,61^ and symptomatic adults and children^62,63^.

Previous studies have found anti-norovirus responses to increase in reactivity and avidity with age, probably due to recurrent infections^26^. In our study, the norovirus infection history of the children is unknown. It is, therefore, not possible to discriminate between cross-reactivity as a result of either multiple norovirus infections or cross-reactivity due to antibodies directed against broadly reactive epitopes. To increase the specificity, assays that measure binding-blocking antibodies could be used^32,64^. Nevertheless, the broad response we detected using antigens representing a large set of genotypes indicates that without knowledge of previous norovirus infections, conclusions about genotype-specific antibody responses might be premature if working with a limited set of antigens.

N-term NS proteins are recognized by antibodies and are genetically diverse, and, therefore, suitable candidates to profile ORF1 immune responses. Our data shows that the immune system produces an antibody response against these non-structural proteins, which is in accordance with epitopes discovered in a recently published phage display study^65^. But based on the broad capsid response of the sera with IgG antibodies towards NS proteins, it is likely that we only detected these after multiple infections and not after a primary infection. Primary infections could induce low quantities of antibodies against non-structural proteins, or the antibody response, as with IgA, could quickly wane. In one challenge study, 74.5% developed antibodies against these antigens, and cross-reactivity was detected between genogroup GI and GII^37,38^. However, the potential function of these antibodies and if they play a role in disease outcome warrants further investigation. Other than seroepidemiological studies screening for antibodies against NS proteins has several additional applications. For example, to differentiate between immunity induced by natural infections versus immunity due to vaccination and to broadly screen for norovirus using the more conserved polymerase and the protease NS proteins.

In conclusion, we have described here, to our knowledge, for the first time the seroprevalence against the majority of known human norovirus genotypes, providing valuable information on serological patterns in a cohort of young children against the diversity of noroviruses. Moreover, we describe the presence of antibodies against ORF1 non-structural proteins, whose potential function warrants further investigation. It remains to be further elucidated how the first norovirus infection shapes the immune response and how the antibody profile correlates with subsequent infection protection.

## Materials and methods

### Sera samples

The different sera used in this study are summarized in Table 3. We used four pre-and post-infection sera to validate the protein microarray and to investigate the change in titer pre and post norovirus infection with a known ORF2 genotype. The 120 sera of children from the Erasmus MC were used to test the antigenicity of the ORF1 proteins. The use of the sera for assay validation was approved by the Erasmus MC medical ethical committee (MEC-2013-082 and MEC-2015-075).

**Table 3:** Summary of sera used.

Sera	Origin	Collection date	Age	Number	Antigens
Pre and Post sera^1^	Erasmus MC Hospital, Rotterdam, the Netherlands	2008–2010	17–43 years	4	P particles
Dutch children’s sera		2010–2015	9 months–2 years	120	NS proteins
MERMAIDS study^41^	From 16 secondary or tertiary hospitals in Belgium, Germany, Greece, Italy, Lithuania, Spain, and the UK	Sep 2016–Mar 2019	< 6 years	287	P particles
				247	NS proteins

^1^RT-PCR confirmed norovirus infection with GI.1, GII.3, or GII.4 (Den Haag 2006b or New Orleans 2009).

To study genotype-specific seroprevalence in children, we used sera of 287 children that were part of a cohort hospitalized with an acute respiratory infection and were collected as part of the Multi-centre EuRopean study of MAjor Infectious Disease Syndromes (MERMAIDS) across seven European countries (Table 3)^41^. Information on sample collection and ethical approval has been reported by Kohns Vasconcelos et al*.*^41^. At the time of sera collection, no gastrointestinal symptoms were reported. Informed consent was obtained from all subjects or their legal guardians. We also confirm that all methods were performed in accordance with relevant guidelines and regulations.

Several studies have shown that most children will experience their first norovirus infection between 6 months and 1 year and that reinfection is common at 2 to 5 years^51,63,66^. To visualize seroprevalences at different ages, the sera were divided into four age groups: newborns with maternal antibodies (0–6 months, n = 85), naïve individuals presumed single infection (> 6–12 months, n = 52), individuals with single or recent infection (> 1–1.5 years, n = 65), and individuals with presumed multiple infections (> 1.5–5.5 years, n = 85). For the NS proteins microarray, 247/287 sera were used as some had to be excluded due to problems during antigen spotting (printing of antigens onto the nitrocellulose slides).

### Plasmid constructs

Sequences of genes encoding the P domain and the non-structural (NS) proteins were custom synthesized (Idt, Coralville, IA, USA) (Table 4). For the P domain, the VP1 sequence was used starting after the hinge region, which is at amino acid position 222 in reference strain GII.4 Sydney 2012 (Accession Nr MT232050)^35^. The amino acid sequences of the antigens are listed in the supplementary materials. The P domains were ligated into the pGEX vector and the NS genes into a pCAGGS-6xHis vector. We included human norovirus genotypes described by Vinjé 2015 and GII.23^67^. GII.22 and GIV.1 were omitted due to failure to produce P particles from these genotypes.

**Table 4:** Noroviruses used for antigen production.

Strain	Accession	Protein
**P particles**^**1**^		
GI.1	M87661	P domain
GI.2	KP064095	P domain
GI.3	MZ735697	P domain
GI.4	KT732281	P domain
GI.5	AM263418	P domain
GI.6	AF093797	P domain
GI.7	AJ844469	P domain
GI.8	KJ196298	P domain
GI.9	KF586507	P domain
GII.1	LN854570	P domain
GII.2	AB662902	P domain
GII.3	MZ841819	P domain
GII.4^2^	MT232050	P domain
GII.5	KJ196288	P domain
GII.6	AB039778	P domain
GII.7	KJ196295	P domain
GII.8	AB039780	P domain
GII.9	AY038599	P domain
GII.10	AY237415	P domain
GII.11	AB074893	P domain
GII.12	KP064099	P domain
GII.13	KC662537	P domain
GII.14	GQ856465	P domain
GII.16	GQ856476	P domain
GII.17	KX424646	P domain
GII.18	AY823305	P domain
GII.19	AY823306	P domain
GII.20	AB542917	P domain
GII.21	KR921942	P domain
GII.23	KJ196291	P domain
**NS proteins**^**3**^		
GI.P2	KF306212	p48, p22
GI.P3	KY934262	p48, p22
GI.P4	LN854563	p48, p22
GI.P11	LN854564	p48, p22
GII.P2	LC209463	p48^4^, p22
GII.P4	KY905331	p48, p22
GII.P7	LN854568	p48
GII.P16	LC175468	p48, p22
GII.P17	KT970377	p48^4^
GII.P18	AY823305	p48, p22
GII.P21	LN854569	p48, p22
GII.P31	MT232050	P48, NTPase, VPg, p22, Protease, RdRp, VP1
GII.PNA2	MG706448	p48
GIII.P2	EU794907	p48
GIV.P1	KC894731	p48
GVI.P1	JF781268	p48
GVII.P1	FJ692500	p48
GX.P1	MF373609	p48

^1^Restriction enzymes BamHI and NotI. The P domain starts at amino acid position 222 of the GII.4 Sydney reference genome (MT232050).

^2^GII.4 Sydney 2012.

^3^Unless mentioned otherwise, NotI/XmaI.

^4^NotI/XhoI.

### Expression and purification of P particles

P particles were produced as described before^35^ with some adaptations. P domains were expressed in *E. coli* strain BL21 overnight at room temperature, and expression was induced with 0.5 mM IPTG (Isopropyl β-d-1-thiogalactopyranoside). The recombinant glutathione S-transferase (GST)-P domain fusion protein was purified using GSTrap Fast Flow columns (Merck, Germany). Bacteria were harvested by centrifugation for 15 min at 3000 rpm. The pellet was resuspended in TB buffer (9.1 mM HEPES, 55 mM MgCl_2_, 15 mM CaCl_2_, 250 mM KCl, pH 6.7) and centrifuged for 20 min at 4000×*g* at 4 °C. The pellet was dissolved in 5 ml lysis buffer (PBS, a protease inhibitor (Roche, Switzerland), 1 µl/ml DNAse, 1 mg/ml lysozyme, 10 mM MgCl_2_), and sonicated on ice 6× for 15 s 38–40%. After centrifugation at 30,000×*g* for 20 min, the supernatant was added onto an equilibrated GSTrap column. The fusion protein was eluted from the GST-tag by PreScission cleavage. For this, the column was washed with 10 ml PBS and incubated with 40 µl PreScission in 960 µl PreScission buffer (50 mM Tri HCl, 150 mM NaCl, 1 mM EDTA, 1 mM DTT, pH 7.5) overnight at 4 °C. The column was washed with 3 ml cleavage buffer, and each ml was collected separately. The protein size of each fraction was checked by sodium dodecyl sulfate–polyacrylamide gel electrophoresis (SDS-PAGE) (Supplementary Fig. 3a). For purification, samples were loaded onto a size exclusion column Superdex 200 column (Amersham Biosciences, USA) powered by an AKTA liquid chromatography system with a flow rate of 1 ml/min (Supplementary Fig. 3b). Samples were concentrated with an Amicon Ultra 15 Centrifugal Filter unit 30-kDa, and protein quantity was defined by NanoDrop and silver staining (ThermoFisher, USA) using a BSA standard (Supplementary Fig. 3c).

### Expression and purification of non-structural proteins

Twenty-four hours before transfection, 3 × 10^6^ 293 T cells were seeded in 10-cm plates in Dulbecco’s modified Eagle’s medium (Lonza, Switzerland, supplemented with 10% fetal bovine serum (Sigma, St. Louis, MO, USA), 1× non-essential amino acids (Lonza), 1× PenStrep (Lonza), 1× l-Glutamine (Lonza) and 1× sodium pyruvate (Gibco, USA). Cells were kept at 37 °C with 5% CO_2_. Cells were transfected using polyethylenimine (PEI, Polysciences, USA). A transfection mix contained 10 µg of plasmid in 30 µl of PEI (1 µg/µl,) in 1 ml OptiMem. After 24–36 h, the cells were harvested and pelleted at 3000 rpm for 15 min. The pellet was lysed in 1 ml precipitation buffer (50 mM Tris, 280 mM NaCl, 0.5% NP-40, 0.2 mM EDTA, 2 mM EGTA, 50% glycerol) with 0.1% DTT and protease inhibitor (Roche) for 5 min at 4 °C. After lysis, the samples were centrifuged for 10 min at 15,300 rpm at 4 °C. 200 µl of Ni–NTA resin (Expedeon, UK) were washed three times with 1 ml washing buffer (precipitation buffer with 0.1% DTT) at 1000 rpm. The supernatant was added to the resin and left to bind on a spinning wheel for 2 h at 4 °C. The resin was washed three times, and samples were eluted in 250 µl elution buffer (precipitation buffer with 0.25 mM Imidazole). The presence of proteins was confirmed by SDS-PAGE and Western blot with an anti-His_6_ antibody (1:1000, ThermoFisher, and anti-mouse (1:10,000, ThermoFisher) (Supplementary Fig. 4). Protein quantity was defined by NanoDrop and with silver staining (ThermoFisher) using a BSA standard.

### Norovirus protein microarrays

Antigens were diluted in protein array buffer (Maine Manufacturing, USA) and protease inhibitor (1:500, BioVision, USA). Antigens were diluted to a final concentration of 0.5 µg/µl and 75 ng/µl for the P particles and the non-structural proteins, respectively. Antigens were spotted in duplicates into 24-pad nitrocellulose coated slides (Oncyte avid, Grace bio-labs, USA) using a sciFLEXARRAYER SX and a Pierce Dispense capillary, PDC coating type2 (SCIENION, Germany). Slides were blocked with 125 µl blocking buffer (ThermoFisher) at 37 °C for 1 h. Sera and antibodies were diluted in blocking buffer substituted with 0.1% Tween 20 Surfactant (ThermoFisher). For all washing steps, PBS was used. After blocking, slides were incubated for 1 h at 37 °C with 125 µl of sera in fourfold dilution starting at 1:20 or 1:40, as stated in the text. Slides were washed six times and incubated with goat anti-human IgG, conjugated with Alexa Fluor 647 (1:1300, Jackson Immuno Research) and Cy3-AffiniPure Goat Anti-Human IgA (1:200, Jackson Immuno Research). Slides were washed with PBS, then with water, and dried. Bound dye was quantified using the PowerScanner (TECAN, Switzerland). As positive control, human immunoglobulin G ((100 mg/ml), Privigen, Germany) was used per slide in the same dilutions as the children’s sera.

### Data analysis

Serum titers were defined as the interpolated serum concentration that gives half the response of the concentration–response between the minimum (3000) and maximum (65,536) relative fluorescent unit (RFU) signal^28,68^. Titers below the minimum dilution were set to a value of < 40, and titers above the highest dilution were set to a value of > 2560. Some pre- and post-sera were diluted up to 1:40,960. Variation was assessed by testing an IgG preparation on each slide. Samples tested on slides with a positive control deviating more than one 2-fold dilution step from the mean were not included in the analyses. The rest were corrected with the factor of deviation from the IgG control as described before^28^. Sera with immunodominant responses were defined as sera that had a ≥ 4-fold higher response against a single genotype compared to the other genotypes. The correlation between IgG and IgA titers was calculated using Spearman’s rank correlation coefficient. The heatmaps were generated in R and reordering was based on mean titers per sample.

Figures were made in GraphPad Prism version 9.4.0 for Windows (GraphPad Software, San Diego, California USA, www.graphpad.com).

## Supplementary Information

Supplementary Information.

## Data Availability

The datasets generated during and/or analyzed during the current study are available from the corresponding author upon reasonable request. All DNA sequences can be found on https://www.ncbi.nlm.nih.gov/genbank/: The accession numbers of P particles can be found in Table 4. The p domain of GI.3 and GII.3 were newly sequenced and deposited under accession numbers MZ735697 and MZ841819, respectively.

## References

[1] Pires SM (2015). Aetiology-specific estimates of the global and regional incidence and mortality of diarrhoeal diseases commonly transmitted through food. PLoS ONE.

[2] Kirk MD (2015). World Health Organization estimates of the global and regional disease burden of 22 foodborne bacterial, protozoal, and viral diseases, 2010: A data synthesis. PLoS Med..

[3] Bartsch SM, Lopman BA, Ozawa S, Hall AJ, Lee BY (2016). Global economic burden of norovirus gastroenteritis. PLoS ONE.

[4] Chhabra P (2019). Updated classification of norovirus genogroups and genotypes. J. Gen. Virol..

[5] Tran TNH, Trainor E, Nakagomi T, Cunliffe NA, Nakagomi O (2013). Molecular epidemiology of noroviruses associated with acute sporadic gastroenteritis in children: Global distribution of genogroups, genotypes and GII.4 variants. J. Clin. Virol..

[6] Patel MM (2008). Systematic literature review of role of noroviruses in sporadic gastroenteritis. Emerg. Infect. Dis..

[7] Inns T, Harris J, Vivancos R, Iturriza-Gomara M, O’Brien S (2017). Community-based surveillance of norovirus disease: A systematic review. BMC Infect. Dis..

[8] Qi R (2018). Global prevalence of asymptomatic Norovirus infection: A meta-analysis. EClinicalMedicine.

[9] Kirby AE (2020). Norovirus seroprevalence among adults in the United States: Analysis of NHANES serum specimens from 1999–2000 and 2003–2004. Viruses.

[10] Cubitt WD, Green KY, Payment P (1998). Prevalence of antibodies to the Hawaii strain of human calicivirus as measured by a recombinant protein based immunoassay. J. Med. Virol..

[11] Gabbay YB (1994). Prevalence of antibodies to Norwalk Virus among Amerindians in isolated amazonian communities. Am. J. Epidemiol..

[12] O’Ryan M, Riera-Montes M, Lopman B (2017). Norovirus in Latin America. J. Pediatr. Infect. Dis..

[13] Nakata S (1998). Prevalence of human calicivirus infections in Kenya as determined by enzyme immunoassays for three genogroups of the virus. J. Clin. Microbiol..

[14] Peasey AE (2004). Seroepidemiology and risk factors for sporadic Norovirus/Mexico strain. J. Infect. Dis..

[15] Kobayashi S, Fujiwara N, Takeda N, Minagawa H (2009). Seroepidemiological study of norovirus infection in Aichi Prefecture, Japan. Microbiol. Immunol..

[16] Smit TK, Steele AD, Peenze I, Jiang X, Estes MK (1997). Study of Norwalk virus and Mexico virus infections at Ga-Rankuwa hospital, Ga-Rankuwa, South Africa. J. Clin. Microbiol..

[17] Mesquita JR, Nascimento MSJ (2014). Norovirus GII.4 antibodies in the Portuguese population. J. Infect. Dev. Ctries.

[18] Son H (2013). Seroepidemiology of predominant Norovirus strains circulating in Korea by using recombinant virus-like particle antigens. Foodborne Pathog. Dis..

[19] Lew JF (1994). Detection of Norwalk virus or Norwalk-like virus infections in Finnish infants and young children. J. Infect. Dis..

[20] Pelosi E (1999). The seroepidemiology of genogroup 1 and genogroup 2 Norwalk-like viruses in Italy. J. Med. Virol..

[21] Gray JJ, Jiang X, Morgan-Capner P, Desselberger U, Estes MK (1993). Prevalence of antibodies to Norwalk virus in England: Detection by enzyme-linked immunosorbent assay using baculovirus-expressed Norwalk virus capsid antigen. J. Clin. Microbiol..

[22] Jing Y, Qian Y, Huo Y, Wang LP, Jiang X (2000). Seroprevalence against Norwalk-like human caliciviruses in Beijing, China. J. Med. Virol..

[23] Dimitrov DH (1997). Prevalence of antibodies to human caliciviruses (HuCVs) in Kuwait established by ELISA using baculovirus-expressed capsid antigens representing two genogroups of HuCVs. J. Med. Virol..

[24] Parker SP, Cubitt WD, Jiang XJ, Estes MK (1994). Seroprevalence studies using a recombinant Norwalk virus protein enzyme immunoassay. J. Clin. Virol..

[25] Kulkarni R, Lole K, Chitambar SD (2016). Seroprevalence of antibodies against Ghildren in Pune, India. J. Med. Virol..

[26] Nurminen K (2011). Prevalence of norovirus GII-4 antibodies in Finnish children. J. Med. Virol..

[27] Belliot G (2001). Characterization of capsid genes, expressed in the baculovirus system, of three new genetically distinct strains of “Norwalk-like viruses”. J. Clin. Microbiol..

[28] van Beek J (2016). Comparison of norovirus genogroup I, II and IV seroprevalence among children in the Netherlands, 1963, 1983 and 2006. J. Gen. Virol..

[29] Jiang XI, Wang MIN, Graham DY, Estes MK (1992). Expression, self-assembly, and antigenicity of the Norwalk virus capsid protein. J. Virol..

[30] Rockx B, Baric RS, De Grijs I, Duizer E, Koopmans MPG (2005). Characterization of the homo-and heterotypic immune responses after natural norovirus infection. J. Med. Virol..

[31] Lindesmith LC (2019). Sera antibody repertoire analyses reveal mechanisms of broad and pandemic strain neutralizing responses after human norovirus vaccination. Immunity.

[32] Lindesmith LC (2015). Broad blockade antibody responses in human volunteers after immunization with a multivalent norovirus VLP candidate vaccine: Immunological analyses from a phase I clinical trial. PLoS Med..

[33] Lindesmith LC (2015). Serum immunoglobulin a cross-strain blockade of human noroviruses. Open Forum Infect. Dis..

[34] Gao J (2021). Antigenic diversity of human Norovirus capsid proteins based on the cross-reactivities of their antisera. Pathogens.

[35] Tan M, Hegde RS, Jiang X (2004). The P domain of norovirus capsid protein forms dimer and binds to histo-blood group antigen receptors. J. Virol..

[36] Chachu KA, LoBue AD, Strong DW, Baric RS, Virgin HW (2008). Immune mechanisms responsible for vaccination against and clearance of mucosal and lymphatic Norovirus infection. PLoS Pathog..

[37] Hesse S (2016). Serological responses to a Norovirus nonstructural fusion protein after vaccination and infection. Clin. Vaccine Immunol..

[38] Ajami NJ (2012). Antibody responses to norovirus genogroup GI.1 and GII.4 proteases in volunteers administered Norwalk virus. Clin. Vaccine Immunol..

[39] Matsui SM (1991). The isolation and characterization of a Norwalk virus-specific cDNA. J. Clin. Investig..

[40] Cotten M (2014). Deep sequencing of norovirus genomes defines evolutionary patterns in an urban tropical setting. J. Virol..

[41] Kohns Vasconcelos M (2021). Aetiology of acute respiratory infection in preschool children requiring hospitalisation in Europe-results from the PED-MERMAIDS multicentre case-control study. BMJ Open Respir. Res..

[42] Lindesmith L (2005). Cellular and humoral immunity following Snow Mountain virus challenge. J. Virol..

[43] Lindesmith LC (2020). Host interactions between nonsecretors and human norovirus. CMGH.

[44] Hansman GS (2006). Genetic and antigenic diversity among noroviruses. J. Gen. Virol..

[45] Ludwig-Begall LF, Mauroy A, Thiry E (2018). Norovirus recombinants: Recurrent in the field, recalcitrant in the lab—A scoping review of recombination and recombinant types of noroviruses. J. Gen. Virol..

[46] Parker SP, Cubitt WD, Jiang X (1995). Enzyme immunoassay using baculovirus-expressed human calicivirus (Mexico) for the measurement of IgG responses and determining its seroprevalence in London, UK. J. Med. Virol..

[47] Iturriza-Gomara M, Elliot A, Dockery C, Fleming D, Gray J (2009). Structured surveillance of infectious intestinal disease in pre-school children in the community:‘The Nappy Study’. Epidemiol. Infect..

[48] Lu L (2019). Genetic diversity and epidemiology of genogroup II noroviruses in children with acute sporadic gastroenteritis in Shanghai, China, 2012–2017. BMC Infect. Dis..

[49] Farahmand M (2021). Global prevalence and genotype distribution of norovirus infection in children with gastroenteritis: A meta-analysis on 6 years of research from 2015 to 2020. Rev. Med. Virol..

[50] Rouhani S (2016). Norovirus infection and acquired immunity in 8 countries: Results from the MAL-ED study. Clin. Infect. Dis..

[51] Saito M (2014). Multiple norovirus infections in a birth cohort in a Peruvian periurban community. Clin. Infect. Dis..

[52] Palmeira P, Quinello C, Silveira-Lessa AL, Zago CA, Carneiro-Sampaio M (2012). IgG placental transfer in healthy and pathological pregnancies. Clin. Dev. Immunol..

[53] Gray J (1994). Detection of immunoglobulin M (IgM), IgA, and IgG Norwalk virus-specific antibodies by indirect enzyme-linked immunosorbent assay with baculovirus-expressed Norwalk virus capsid antigen in adult volunteers challenged with Norwalk virus. J. Clin. Microbiol..

[54] Atmar RL (2014). Determination of the 50% human infectious dose for Norwalk virus. J. Infect. Dis..

[55] van Beek J (2018). Molecular surveillance of norovirus, 2005–16: An epidemiological analysis of data collected from the NoroNet network. Lancet Infect. Dis..

[56] Cao R-R (2021). Epidemiology of norovirus gastroenteritis in hospitalized children under five years old in western China, 2015–2019. J. Microbiol. Immunol. Infect..

[57] Chan MC (2015). Virus genotype distribution and virus burden in children and adults hospitalized for norovirus gastroenteritis, 2012–2014, Hong Kong. Sci. Rep..

[58] Chen H (2015). A novel norovirus GII.17 lineage contributed to adult gastroenteritis in Shanghai, China, during the winter of 2014–2015. Emerg. Microbes Infect..

[59] Haddadin Z (2020). Characteristics of GII.4 Norovirus versus other genotypes in sporadic pediatric infections in Davidson County, Tennessee, USA. Clin. Infect. Dis..

[60] Phattanawiboon B (2020). Norovirus transmission mediated by asymptomatic family members in households. PLoS ONE.

[61] García C, DuPont HL, Long KZ, Santos JI, Ko G (2006). Asymptomatic norovirus infection in Mexican children. J. Clin. Microbiol..

[62] Bruggink LD, Dunbar NL, Marshall JA (2015). Norovirus genotype diversity in community-based sporadic gastroenteritis incidents: A five-year study. J. Med. Virol..

[63] Chhabra P (2020). Homotypic and heterotypic protection and risk of re-infection following natural norovirus infection in a highly endemic setting. Clin. Infect. Dis..

[64] van Loben Sels JM (2021). A luciferase-based approach for measuring HBGA blockade antibody titers against human norovirus. J. Virol. Methods.

[65] Huang W (2020). High-resolution mapping of human norovirus antigens via genomic phage display library selections and deep sequencing. J. Virol..

[66] Cannon JL, Lopman BA, Payne DC, Vinjé J (2019). Birth cohort studies assessing norovirus infection and immunity in young children: A review. Clin. Infect. Dis..

[67] Vinje J (2015). Advances in laboratory methods for detection and typing of Norovirus. J. Clin. Microbiol..

[68] Koopmans M (2012). Profiling of humoral immune responses to influenza viruses by using protein microarray. Clin. Microbiol. Infect..

