# Supplementary material for: Profiling of humoral immune responses to norovirus in children across Europe
Source: Sci Rep. 2022 Aug 22;12:14275. doi: 10.1038/s41598-022-18383-6 (PMC9395339; doi:10.1038/s41598-022-18383-6)
Supplement: Supplementary file 1 — Supplementary Information. [file 41598_2022_18383_MOESM1_ESM.pdf]

Profiling of humoral immune responses to norovirus in children across Europe

Nele Villabruna[1], Ray W. Izquierdo-Lara[1], Claudia M.E. Schapendonk[1], Erwin de Bruin[1], Felicity Chandler[1], Tran Thi Nhu Thao[2,3,4], Brenda M. Westerhuis[1], Janko van Beek[1], Louise Sigfrid[5], Carlo Giaquinto[6], Herman Goossens[7], Julia A. Bielicki[8,9], Malte Kohns Vasconcelos[8,10], Pieter L.A. Fraaij[11], Marion P.G. Koopmans[1], Miranda de Graaf[1]*

Supplementary information

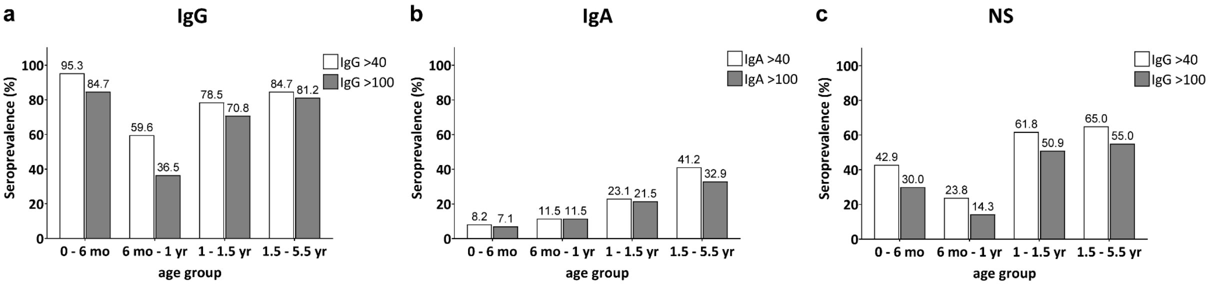

Supplementary Figure 1. Norovirus seroprevalence in four age groups. Seroprevalence is calculated with a cutoff of >40 and >100 for titers to be considered positive **a)** IgG, **b)** IgA, and **c)** IgG against NS proteins. Mo=months, yr=years

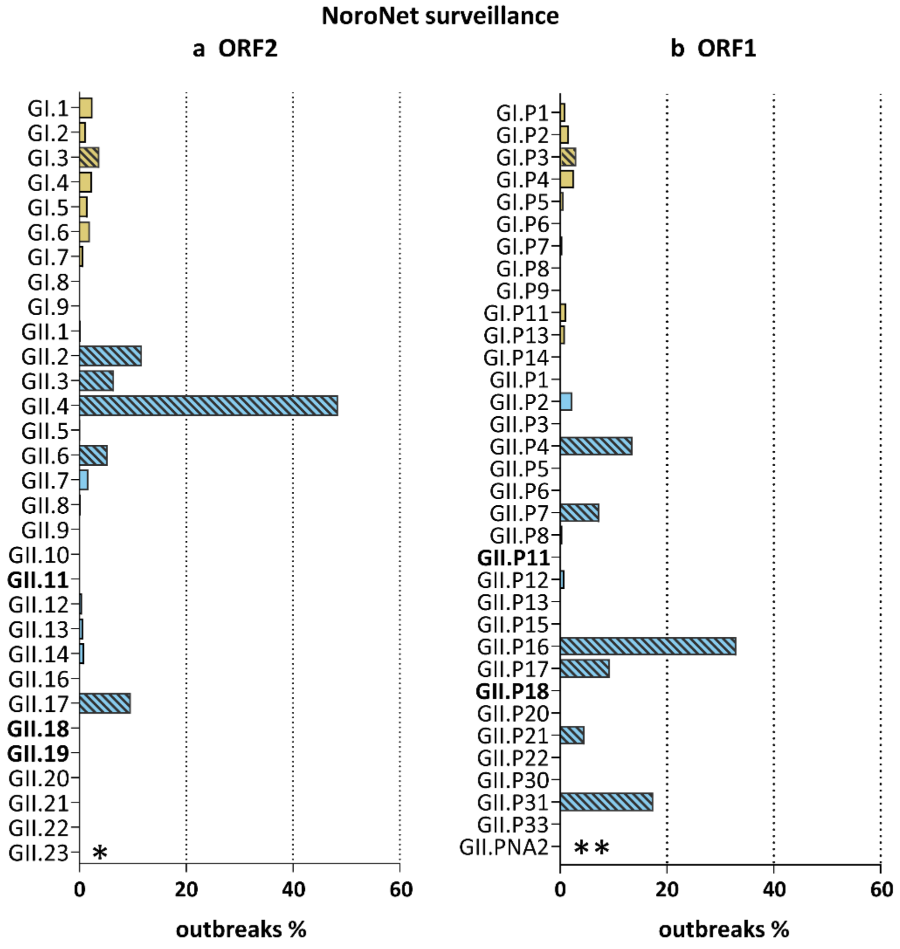

Supplementary Figure 2. NoroNet data from outbreaks in Europe between 2016 and 2019. Prevalence of **a)** ORF2 and **b)** ORF1 genotypes detected in outbreaks. *GII.23 is not listed in NoroNet as a genotype yet. **GII.pNA2 is not included in the NoroNet database. The genotypes most commonly reported during outbreaks are marked with a pattern. In bold are the porcine genotypes. The numbers of outbreaks for each ORF1 and OR2 genotypes were extracted separately from NoroNet with a sample collection date between 1.1.2016 and 31.12.2019. Data were extracted on 8.7.2021.

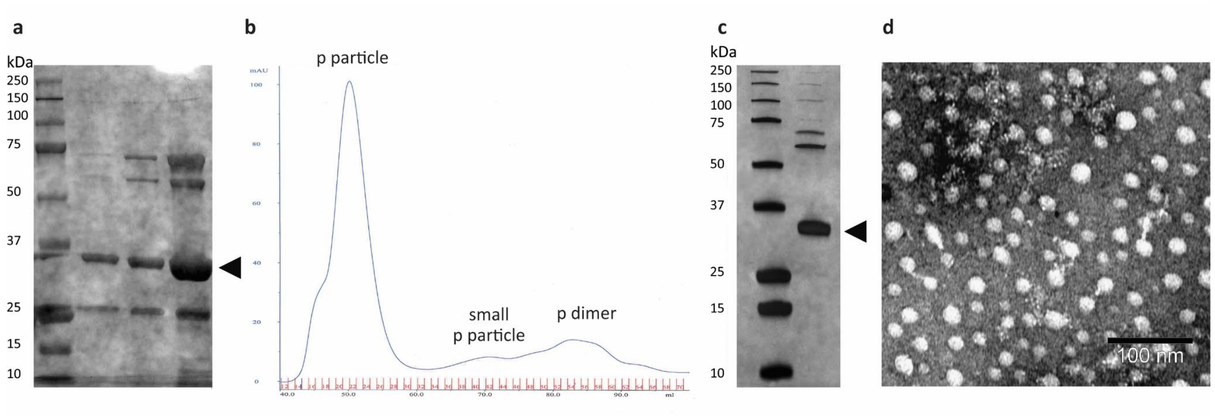

Supplementary Figure 3. P particle expression and purification. a) GST-tagged fusion proteins were cleaved with PreScission and the fractions were collected. **b)** samples were purified by high performance liquid chromatography and the fraction with the peak representing the P particles, were collected. **c)** Samples were concentrated and added on SDS-PAGE gel for quantification. **d)** Analysis of P particles by electron microscopy. **Black arrow heads** show the p domain at its estimated size of 35 kDa. Original gels are presented in **Supplementary Figure 5**.

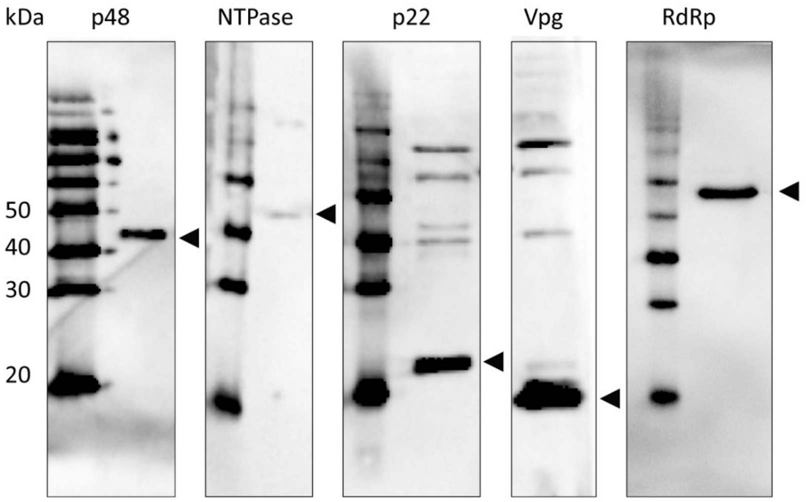

Supplementary Figure 4. Expression of non-structural proteins. His-tagged proteins were expressed in 293T cells and purified using Ni-NTA resin. The presence of proteins was confirmed by Western blot with an anti-His6 antibody (MA1-21315). **Black arrow heads** show the ns proteins. Original blots are presented in **Supplementary Figure 5**.

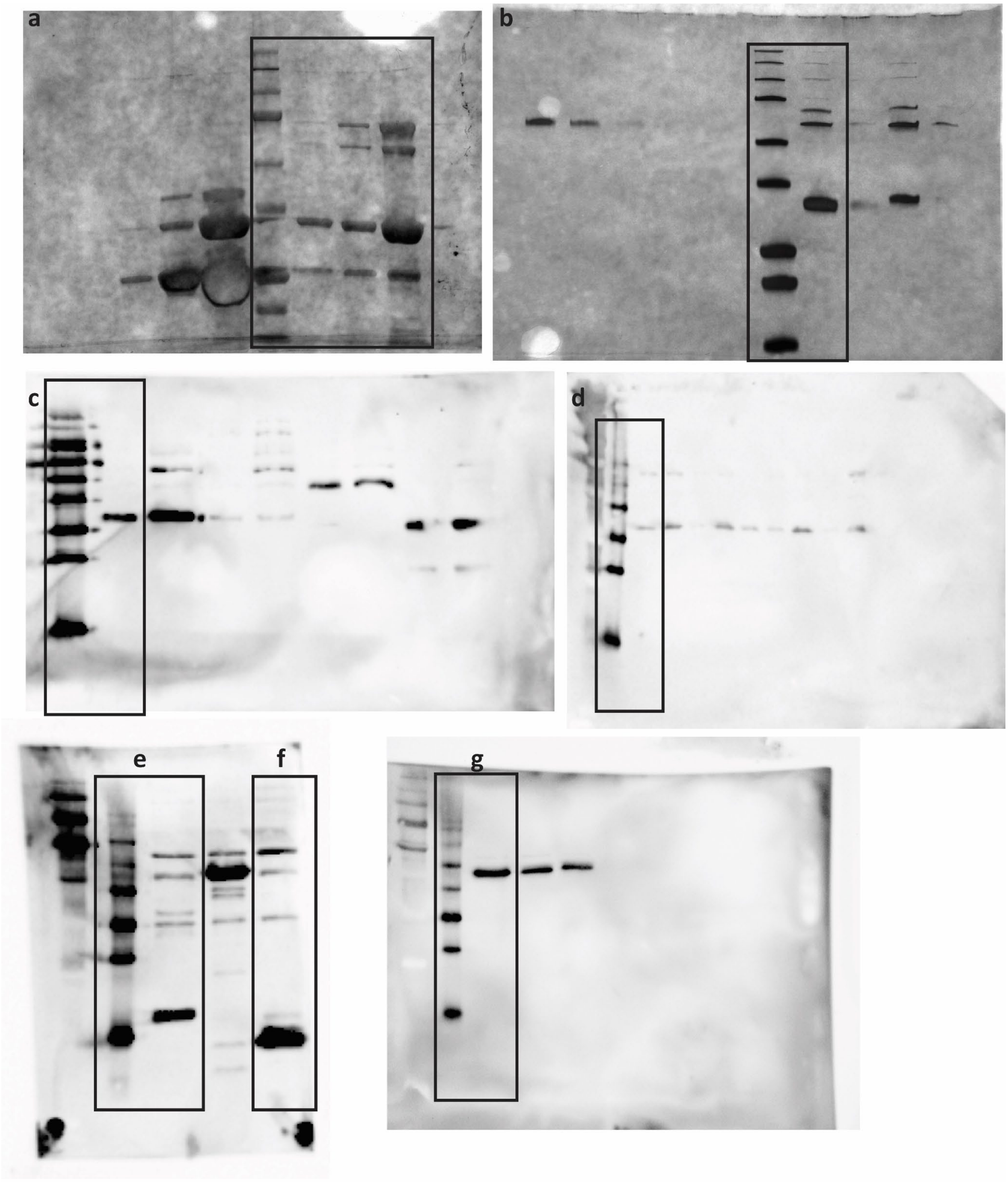

Supplementary Figure 5. Original SDS-page gels and Western blots. The rectangles mark the excerpt presented in **a)** Supplementary Figure 3a, **b)** Supplementary Figure 3c, and Supplementary Figure 4 **c)** p48 **d)** NTPase **e)** p22 **f)** VPg and **g)** RdRp.

Supplementary Table 1. IgG titers against NS proteins in pre- and post-infection sera. Marked in **red** are the titers that were >4-fold increased in post-infection sera compared to pre-infection sera. IgA titers are not shown as they were all negative.

			GI.P2	GI.P3	GI.P4	GI.P11	GII.P2	GII.P4	GII.P7	GII.P16	GII.P17	GII.P18	GII.P21	GII.P31	GII.PNA2	GIII.P2	GIV.P1	GVI.P1	GVII.P1	GX.P1
GI.1	p48	Pre	<40	<40	<40	<40	<40	<40	<40	<40	<40	<40	<40	<40	<40	<40	<40	<40	<40	<40
		Post	<40	<40	<40	<40	<40	<40	<40	<40	<40	<40	<40	<40	<40	<40	<40	<40	<40	<40
		fold increase	0	0	0	0	0	0	0	0	0	0	0	0	0	0	0	0	0	0
	p22	Pre	<40	<40	<40	<40	<40	<40	NA	<40	NA	<40	<40	<40	<40	NA	NA	NA	NA	NA
		Post	<40	<40	<40	<40	<40	<40	NA	<40	NA	<40	<40	<40	<40	NA	NA	NA	NA	NA
		fold increase	0	0	0	0	0	0	0	0	0	0	0	0	0					
GII.3	p48	Pre	<40	<40	<40	<40	<40	75	<40	<40	<40	<40	<40	64	<40	<40	<40	<40	<40	<40
		Post	<40	<40	<40	<40	<40	88	<40	<40	<40	<40	<40	81	41	<40	<40	<40	<40	<40
		fold increase	0	0	0	0	0	1.2	0	0	0	0	0	1	1	0	0	0	0	0
	p22	Pre	<40	<40	<40	<40	<40	<40	NA	<40	NA	<40	<40	<40	<40	NA	NA	NA	NA	NA
		Post	<40	<40	<40	<40	<40	<40	NA	<40	NA	<40	<40	<40	<40	NA	NA	NA	NA	NA
		fold increase	0	0	0	0	0	0	0	0	0	0	0	0	0					
GII.4 Den Haag 2006b	p48	Pre	<40	<40	<40	<40	<40	105	51	<40	<40	<40	52	44	<40	<40	<40	<40	<40	<40
		Post	<40	<40	<40	<40	<40	6039	121	<40	351	<40	1607	854	978	<40	<40	<40	<40	<40
		fold increase	0	0	0	0	0	57.5	2	0	9	0	40.2	19.6	24.4	0	0	0	0	0
	p22	Pre	<40	<40	<40	<40	<40	<40	NA	<40	NA	<40	<40	<40	<40	NA	NA	NA	NA	NA
		Post	<40	<40	<40	<40	<40	<40	NA	<40	NA	50	61	134	<40	NA	NA	NA	NA	NA
		fold increase	0	0	0	0	0	0	0	0	0	1.3	1.5	3.4	0					
GII.4 New Orleans	p48	Pre	<40	<40	<40	<40	<40	<40	<40	<40	NA	<40	<40	<40	<40	NA	NA	NA	NA	NA
		Post	<40	<40	<40	<40	<40	342	<40	<40	NA	<40	<40	<40	<40	NA	NA	NA	NA	NA
		fold increase	0	0	0	0	0	8.5	0	0	0	0	0	0	0	0	0	0	0	0
	p22	Pre	<40	<40	<40	<40	<40	<40	NA	<40	NA	<40	<40	<40	<40	NA	NA	NA	NA	NA
		Post	<40	<40	<40	<40	<40	<40	NA	<40	NA	<40	<40	<40	<40	NA	NA	NA	NA	NA
		fold increase	0	0	0	0	0	0	0	0	0	0	0	0	0					

Antigen sequences

P domains

GI.1
>M87661 Norwalk virus
QKTRPFTLPNLPLSSLSNSRAPLPISSMGISPDNVQSVQFQNGRCTLDGRLVGTTPVSLSHVAKIRGT
SNGTVINLTELDGTPFHPFEGPAPIGFPDLGGCDWHINMTQFGHSSQTQYDVDTTPDTFVPHLGSIQA
NGIGSGNYVGVLSWISPPSHPSGSQVDLWKIPNYGSSITEATHLAPSVYPPGFGEVLVFFMSKMPGPG
AYNLPCLLPQEYISHLASEQAPTVGEAALLHYVDPDTGRNLGEFKAYPDGFLTCVPNGASSGPQQLPI
NGVFVFVSWVSRFYQLKPVGTASSARGRLGLRR

GI.2
>KP064095 Norovirus GI.2
QKTRAFTVPNIPLQTLSNSRFPSLIQGMILSPDASQVVQFQNGRCLIDGQLLGTTPATSGQLFRVRGK
INQGARTLNLTEVDGKPFMAFDSPAPVGFPDFGKCDWHMRISKTPNNTSSGDPMRSVSVQTNVQGFVP
HLGSIQFDEVFNHPTGDYIGTIEWISQPSTPPGTDINLWEIPDYGSSLSQAANLAPPVFPPGFGEALV
YFVSAFPGPNNRSAPNDVPCLLPQEYITHFVSEQAPTMGDAALLHYVDPDTNRNLGEFKLYPGGYLTC
VPNGVGAGPQQLPLNGVFLFVSWVSRFYQLKPVGTASTARGRLGVRRI

GI.3
>MZ735697 Norwalk virus
QKTRPFTLPNLPLSSLSNSRAPLPISSMGISPDNVQSVQFQNGRCTLDGRLVGTTPVSLSHVAKIRGT
SNGTVINLTELDGTPFHPFEGPAPIGFPDLGGCDWHINMTQFGHSSQTQYDVDTTPDTFVPHLGSIQA
NGIGSGNYVGVLSWISPPSHPSGSQVDLWKIPNYGSSITEATHLAPSVYPPGFGEVLVFFMSKMPGPG
AYNLPCLLPQEYISHLASEQAPTVGEAALLHYVDPDTGRNLGEFKAYPDGFLTCVPNGASSGPQQLPI
NGVFVFVSWVSRFYQLKPVGTASSARGRLGLRR

GI.4
>KT732281 Norovirus GI.4
QKTRPFTVPNIPLKYLSNSRIPNPIEGMSLSPDQTQNVQFQNGRCTIDGQPLGTTPVSVSQLCKFRGR
ITSGQKVLNLTELDGSPFMAFAAPAPAGFPDLGSCDWHIEVSKIPNSSTQGNPIVVNSVKPNSQQFVP
HLSSITLDDNVSSGGDYIGTIQWTSPPSDSGGANTNFWKIPDYGSSLAEASQLAPAVYPPGFNEVIVY
FMASIPGPNQSGSPNLVPCLLPQEYITHFISEQAPIQGEAALLHYVDPDTNRNLGEFKLYPGGYLTCV
PNSSSTGPQQLPLDGVFVFASWVSRFYQLKPVGTAGPARGRLGVRR

GI.5
>AM263418 Norovirus Hu/GI/Babbacombe/1996/GBR
QKTRPFSVPNIPLQLLSNSRVPNLIQSMVLSPDQAQNVQFQNGRCTTDGQLLGT
TPVSVSQILKFRGKVSAGSRVINLTELDGSPFLAFEAPAPTGFPDLGTSDWHVEMSLNSNSQSSGNPI
LLRDIQPNSSDFVPHLGSVAVTTAIDVAGDYTGTIQWTSQPSNVTPVPDVNFWTIPQYGSNLAEASQL
APVVYPPGFGEAIVYFMSPIPGPNTAHKPNLVPCLLPQEFVTHFVSEQAPSMGEAALVHYVDPDTNRN
LGEFKLYPEGFITCVPNGTGPQQLPLNGVFVFASWVSRFYQLKPVGTASSARGRLGVRR

GI.6
>AF093797 Norwalk virus
QKTRVFSVPNIPLKDLSNSRVPTLIQGMFVSPDVNQSVQFQNGRCQIDGQLQGTTPVSLSQLCKIRGR
TSSNTRVLNLSEVDGTPFVPLESPAPVGFPDIGGCDWHVGFTFEARDQDPSQNVTFATNDSSFVPYLG
SISPHNGDGFHSGDIIGSLDWISAPSDGSALDVWSIPKYGSSLPDVTHLAPAVFPPGFGEVILYFHSK
FPGSGPTDKLRVPCLIPQEFITHFCNEQAPIAGEAALLHYVDPDTGRNLGEFKLYPDGFMTCVPNSIS
SGPQTLPINGVFVFVSWVSRFYQLKPVGTASAARRLGLRRI

GI.7

>AJ844469 Hu/NV/Chiba/030100/2003/JP

QKTRQLTVPNIPLNNLSNSRVPAMISKMAISSDANQVVQFQNGRCTTDGQLLGTTPISASQVARIRGK
VFSTSSGKGLNLSELDGSPYHAFESPAPVGFPDLGYCDWHVSSFKADHNSSSDPISRLDIKQGASFAP
HLGSIEYTTSQNPDGDQLGTLTWISSPSDDTPGHGTINLWKIPSYGSTVTESVHLAPPIYPPGFGETL
VYFMSDFPIGHTNSNMAQVPCTLPQEFVAHFVNEQAPVRGDAALLHYVDPDTHRNLGEFKLYPEGYIT
CVPNTGGGGPQSLPINGVFIFSSWVSRYYQLKPVGTAGPARRLGVRRV

GI.8

>KJ196298 Norovirus GI/Hu/JP/2007/GI.P8_GI.8/Nagoya/KY531

QRTKPFSVPNIPMNLMSNSRVSMLIDGMMVSNDQNQVPQFQNGRVTLDGQLQGTTTVSAACVARMRGR
IFNNNGNYGVNLTELDGNPYHAFDSPAPLGFPDFGNCDLHMTFVKINPNELSSGDPSGKVVIHSYDAT
FAPHLGTVKLENDDELARFVGKEVVLELTWVSNREGATLNLWAVPNYGSSLTQASQLAPPIYPPGFGE
AIVYFTSTFPTVSNPKVPCTLPQEFVSHFVNEQAPTRGDAALLHYVDPDTHRNLGEFKMYPEGYMTCV
PNAGGGPQTLPINGVFVFISWVSRYYQLKPVGTAGAARRLGLRRS

GI.9

>KF586507 Norovirus GI.9

QKTKQFSVPNLPLNVMSNSRVPSLLNAMVVSPDQAQVVQFQNGRCTLDGQMLGTTTVSASCVARFRGK
TFQAPDNRLGINLAEISGEPYHAFESPAPLGFPDFGDGDWHVTATKVTPSQLEANDPVVMGNVQPYNP
QFAPHLGTLVVENPTPDNVTTGTDLLFNITWLSNRANNRFNPWVIPNYGSTLTEAAQLAPSIFPPGFG
ETIVYFNSTFPAVGATTHAAIPCLLPQEFVAHFVNEQAPIRGEAALLHYIDPDTHRNLGEFKIYPEGF
VTCVPNVGGTGPQSLPTNGIFVFVSWVSRYYQLKPVGTAGQARRLGFRRV

GII.1

>LN854570 GII/Hu/NL/2014/GII.2/Groningen

SKTKPFTLPILTIGELSNSRFPVPIDILYTSPNEGVVVQPQNGRSTLDGELLGTTQIVPSNICALRGR
INAHVPNEPNQWNLQVTNANGTPFDPTEDVPAPLGTPDFQANIYGVTGQRNSDSSCRAHDGVLATWSP
KFTPKLGSVVLGTWEKEDFNINQPTRFTPIGLHNTEHFKQWVLPNYSGMLTLNMNLAPSVAPLFPGEQ
ILFFRSFIPLNGGKSDGAIDCLLPQEWIQHFYQESAPSPTDVALIRYTNPDTGRVLFEAKLHRQGFIT
VASSGSKPIVVPPNGYFRFDSWVNQFYSLAPMGTGNGRRRVQ

GII.2

>AB662902 Norovirus Hu/GII.2/OH10031/2010/JP

SKTKPFTLPILTLGELSNSRFPVSIDQMYTSPNEVISVQCQNGRCTLDGELQGTTQLQVSGICAFKGE
VTAHLHDNDHLYNITITNLNGSPFDPSEDIPAPLGVPDFQGRVFGIISQRDKHNSPGHNEPANRGHDA
VVPTYTAQYTPKLGQIQIGTWQTDDLTVSQPVKFTPVGLNDTEHFNQWVVPRYAGALNLNTNLAPSVA
PVFPGERLLFFRSYIPLKGGYGNPAIDCLLPQEWVQHFYQEAAPSMSEVALVRYINPDTGRALFEAKL
HRAGFMTVSSNTSAPVVVPANGYFRFDSWVNQFYSLAPMGTGNGRRRVQ

GII.3

>MZ841819 Norovirus GII

SKTKPFSLPILTISEMSNSRFPVPIDSLHTSPTENIVVQCQNGRVTLDGELMGTTQLLPSQICAFRGV
LTRSTSRASDQTDTATPRLFNYYWHIQLDNLNGTPYDPAEDIPGPLGTPDFRGKVFGVASQRNPDATT
RAHEAKIDTTSGRFTPKLGSLEISTESSDFDQSQPTRFTPVGIGVDHEADFQQWTLPDYAGQFTHNMN
LAPAVAPNFPGEQLLFFRSHLPSSGGRSNGILDCLVPQEWVQHFYQESAPSQSQVALVRYVNPDTGRV
LFEAKLHKLGFMTIAKNGDSPITVPPNGYFRFESWVNPFYTLAPMGTGNGRRRIQ

GII.4

>MT23205 Norovirus GII

SRTKPFSVPVLTVEEMTNSRFPIPLEKLFTGPSSAFVVQPQNGRCTTDGVLLGTTQLSPVNICTFRGD
VTHITGSRNYTMNLASQNWNNYDPTEEIPAPLGTPDFVGKIQGVLTQTTRTDGSTRGHKATVYTGSAD
FAPKLGRVQFETDTDHDFEANQNTKFTPVGVIQDGSTTHRNEPQQWVLPSYSGRNTPNVHLAPAVAPT

FPGEQLLFFRSTMPGCSGYPNMDLDCLLPQEWVQYFYQEAAPAQSDVALLRFVNPDTGRVLFECKLHK
SGYVTVAHTGQHDLVIPPNGYFRFDSWVNQFYTLAPMGNGTGRRRAL

GII.5
>KJ196288 Norovirus GII/Hu/JP/2002/GII.P5_GII.5/Saitama/T52
SKTKPFTLPVLTLGELSNSRFPLSIDEMVTSPNESIVVQPQNGRVTLDGELLGTTQLQACNICSIRGK
VTGQVPNEQHMWNLQITNLNGTQFDPTDDVPAPLGVPDFAGEVFGVLSQRNRGESNPANRAHDAVVAT
YSDKYTPKLGLVQIGTWNTNDVENQPTKFTPIGLNEVANGHRFEQWTLPRYSGALTLNMNLAPAVAPL
FPGERLLFFRSYVPLKGGFGNPAIDCLVPQEWVQHFYQESAPSLGDVALVRYVNPDTGRVLFEAKLHK
GGFLTVSSTSTGPVVVPANGYFRFDSWVNQFYSLAPMGTGNGRRRFQ

GII.6
>AB039778 Norwalk-like virus
SKTKPFTLPILTLGELSNSRFPAPIDMLYTDPNETIVVQPQNGRCTLDGTLQGTTQLVPTQICSFRGT
LISQTSRSADSTDSAPRVRNHPLHVQLKNLDGTPYDPTDEVPAVLGAIDFKGTVFGVASQRNTTGSSV
GATRAHEVHIDTTNPRYTPKLGSVLMYSESSDFDDGQPTRFTPIGMGADDWHQWELPEYSGHLTLNMN
LAPAVAPAFPGERILFFRSVVPSAGGYGSGQIDCLIPQEWVQHFYQEAAPSQSAVALIRYVNPDTGRN
IFEAKLHREGFITVANSGNNPIVVPPNGYFRFEAWVNQFYTLTPMGTGQGRRRVQ

GII.7
>KJ196295 GII/Hu/JP/2010/GII.P7_GII.7/Musashimurayama/TAKAsanKimchi
SKTKQFTLPILKISEMTNSRFPVPVEMMYTARNENQVVQPQNGRVTLDGELLGTTPLLAVNICKFKGE
VIAKNGDVRSYRMDMEITNTDGTPIDPTEDTPGPIGSPDFQGILFGVASQRNKNEQNPATRAHEANIN
TGGDQYAPKLAQVKFFSESQDFEVHQPTVFTPVGVAGDTSHPFRQWVLPRYGGHLTNNTHLAPAVAPL
FPGEQILFFRSQIPSSGGHELGYMDCLVPQEWVQHFYQEAATAQSEVALIRFINPDTGRVLFEAKLHK
QGFITVAHTGDNPIVMPPNGYFRFEAWVNQFYSLAPVGTGNGRRRIQ

GII.8
>AB039780 Norwalk-like virus
SKTKAFTLPILKISEMTNSRFPIPVDQMYTSRNENIVVQPQNGRVTLDGELQGTTTLQPVSICGFRGT
LQTRLADQPNYTYQVHLENLDGSPVDPTDEVPAPLGTPDFQAQLFGVISQRSSDNATRAHEARVNTND
PTFAPQIAQVRFKSPSNDFFDNEPIKFTPVGISVDSQNSYNQWLLPRYGGHLTNNTHLAPSVSPMFPG
EQILFFRSFMPGASGHTDGAIDCLLPQEWVAHFYQEAATAQTDVALIRFVNPDTGRVLFEGKLHKQGF
ITISNSGDHPIVMPANGYFRFEAWVNQFYSLAPVGTGSGRRRIQ

GII.9
>AY038599 NLV/VA97207/1997
SKTKAFTIPVLKISEMTNSRFPVPVDQMYTSRSEGIVVQPQNGRATIDGELLGTTLVSPVSVCNFKGN
LQAEVPGQHQLYQLQLTNLDGSPIDPTDDTPGPLGCPDFTGLLYGVASQRGPGDATRAHEARIDTGSD
TFAPKIGQVRFYSTSSDFETNQPTHFTPIGIYIEGNSSDFNQWQLPRYGGHLANNNHLAPAVSPLFPG
EQILFFRSFIPGASGHTNGEMDCLLPQEFVQHFYQEAATARSEVALLRFVNPDTGRALFESKLHKQGF
MTIASSGDHPIIMPTNGYFRFEAWVNQFYSLAPVGTGSGRRRIQ

GII.10
>AY237415 Human calicivirus strain Mc37
SKSKPFTLPILTLGELTNSRFPLPIDVLYTNPNESAIVQCQNGRCTLDGELQGTTQLLPTGICAFRGK
VTQQVQDEHRGTHWNMTVTNLNGTPFDPTEDVPAPLGTPDFSGQIYGVISQRNTNTVPGEGNLPANRA
HEAVIATYSPKFTPKLGNIQFSTWETQDVSSGQPTKFTPVGLASVDANSHFDQWTLPSYSGALTLNMN
LAPSVAPVFPGECLLFFRSFIPLKGGYGNPAIDCLMPQEWVQHLYQESAPSLSDVALVRYVNPETGRT
LFEAKLHRNGFLTVARNSAGPVVAPTNGYFRFDSWVNQFYTLAPMGNGSGRRRMQ

GII.11
>AB074893 Swine norovirus
SKTKPFSLPGLTLDELSNSRFPAPIVQLYTNPHDNLIVQPQNGRCTIDGLLQGTTQLVSCNVCSFRGT
MGEGQPAAHSEDEIMPMAFNVQREIMLENLDGSPYDPTDDIPAVLGSPDFQGVMFGILSQRNTDGQTR
AHEAKVDTRAARFAPKLGFVVATVESTDFHANQPSRFTPVGLGGDTNRDFNQWQLPAYGGALTNNTNL
APPVMPVYPGEQLLFFRSQLPSSGGVVAGWLDCLLPQEWVQHFFQESATSQSDVALVRYVNPTTGRVL
FEAKLHKQGFLTVAASGSYPLVVPADGYFRFESWVNQFYTLAPMGNGSGRRRAR

GII.12
>KP064099 Norovirus GII.12
SKTKPFTLPILTIGELTNSRFPVPIDELYTSPNESLVVQPQNGRCALDGELQGTTQLLPTAICSFRGR
INQKVSGDNHVWNMQVTNINGTPFDPTEDVPAPLGTPDFSGKLFGVLSQRDHDNACRSHDAVIATDSA
KFTPKLGAIQIGTWEEDDVHINQPTKFTPVGLFEREGFNQWTLPNYSGALTLNMGLAPPVAPTFPGEQ
ILFFRSHIPLKGGVADPVIDCLLPQEWIQHLYQESAPSQTDVALIRFTNPDTGRVLFEAKLHRSGYIT
VANTGSRPIVVPANGYFRFDSWVNQFYSLAPMGTGNGRRRVQKG

GII.13
>KC662537 Norovirus Hu/GII/Hy-718/KOR
SKTKPFTLPILTISELTNSRFPIPIEQLYTAPNETNVVQCQNGRCTLDGELQGTTQLLSSAVCSYRGR
TVANNGDNWDQNLLQLTYPNGASYDPTDEVPAPLGTQDFSGMLYGVLTQDNVNVSTGEAKNAKGIYIS
TTSGKFTPKIGSIGLHSITEHVHPNQQSRFTPVGVAVNENTPFQQWVLPHYAGSTRSHTNLGTALAPT
FPGEQLLFFRSRVPCVQGLQGQDAFIDCLLPQEWVNHFYQEAAPSQADVALIRYVNPDTGRTLFEAKL
HRSGFITVSHTGAYPLVVPPNGHFRFDSWVNQFYSLAPMGTGNGRRRIQ

GII.14
>GQ856465 Hu/GII.4/Beijing/55028/2007/CHN
SKTKNFTLPVLRVSEMTNSRFPVVLDQMYTSRNENIIVQPQNGRCTTDGELLGTTILQSVSICNFKGT
MQAKLNEEPRYQLQLTNLDGSPIDPTDDMPAPLGTPDFQAMLYGVASQRSSTDNATRAHDAQIDTAGD
TFAPKIGQVRFKSSSNDFDLHDPTKFTPIGVNVDDQHPFRQWSLPNYGGHLALNNHLAPAVTPLFPGE
QILFFRSYIPSAGGHTDGAMDCLLPQEWVEHFYQEAAPSQSDIALVRFINPDTGRVLFEAKLHKQGFL
TIAASGDHPIVMPTNGYFRFEAWVNPFYTLAPVGTGSGRRRIQ

GII.16
>GQ856476 Hu/GII.4/Beijing/55171/2008/CHN
SKTKPFSVPVLTLNELTNSRFPVPIDAMYTSPNDSIVVQPQNGRATIDGELLGTTQLIPSGICSFRGK
ITTHLADDRHLWNIQVSNLNGTPFDPTDDVPAPLGMPDFSGQIFGVVSQRDTGTNPANRAHDAVLATY
SAKYTPKLGSVQIGTWDTEDLLERQPVKFTPVGLNEIGQDKHFDQWVLPNYSGALGLNMHLAPAVSPL
FPGERLLFFRSYIPLKGGHGDPFIDCLVPQEWIQHFYQESAPAQSSVALLRYVNPDTGRTLFEAKLHK
GGFITVSSTENRPVIVPPNGYFRFDSWVNQFYSLAPMGTGNGRRRMQ

GII.17
>KX424646 Norovirus GII
SKTKPFSLPILTLSELTNSRFPVPIDSLFTAQNNVLQVQCQNGRCTLDGELQGTTQLLPSGICAFRGR
VTAQINQRDRWHMQLQNLNGTTYDPTDDVPAPLGTPDFKGVVFGMVSQRNVGNDAPGSTRAQQAWVST
YSPQFVPKLGSVNLRISDNDDFQFQPTKFTPVGVNDDDDGHPFRQWELPNYSGELTLNMNLAPPVAPN
FPGEQLLFFRSFVPCSGGYNQGIIDCLIPQEWIQHFYQESAPSQSDVALIRYVNPDTGRTLFEAKLHR
SGYITVAHSGDYPLVVPANGHFRFDSWVNQFYSLAPMGTGNGRRRAQ

GII.18
>AY823305 swine/GII/OH-QW125/03/US
SKSKPFTIPQLTLSELSNSRFPAPIDMLYTSPNDNIVVQPQNGRCTLGGELQGTTQLQPSLICSFRGV
TLNETSRMLVDQEEEEEGEVQRPQARAFNHEYHLQLQNLDGTDYDPAGDLPATLGAPDFIGTVYGVVS
QRTTRDSAARAHEAKITTSDPRYAPKLGSVEFVTESTDIVLNDQTKFTPVGLAHNRAVDYKQWELPHY

AGELTANTNLAPPVAPVYPGEQLLFFRSNLPLAGGTAASVLDCLLPQEWIQHFYQESFAPQSDIALLR
YVNPDTGRVLFEAKLHRQGYITVAGAGTSPVAVPPNGYFRFESWVSQFYTLAPMGSGTGRRRNQ

GII.19
>AY823306 swine/GII/OH-QW170/03/US
SKTKPFSLPNLTLAELSNSRFPLPIEQLYTNPLDNLVVQPQNGRCTIDGILLGTTQLVPSLICSMRGT
LEADQASGHTDDESVQSRAFNRQRSLMLENPDGSAFDPSDEVPAVLGVPDFRGVVFGVLSQRNNGDTT
RSHEAKIDTRRPRYAPKLGFVEVLTESDDFQNGPPTKFTPVGLGGDGNPDFQQWALPDYGGALTNNTN
LAPPVVPVYPGEQILFFRSQLPSSGGTVAGWLDCLLPQEWIQHFYQDSAAAQSDVALVRYINPATGRV
LFEAKLHRQGFLTVSAAGPHPVVAPADGYFRFESWVNQFYTLAPMGNGSGRRRVY

GII.20
AB542917 Hu/OC07118/2007/JP
SKSKPFTIPMLTIEEMTNSRFPAPLELMTTGPSHDIVVQPQNGRCTIDGVLLGTTQLSPVNICSLRGV
PTKRTNGNDNCFVQLENPNGSAYDPTEDIPAVLGSPDFVGELYGTITQRSSDNSTRAHPFTLNTGSPR
YTPKIGSVDIRVTDVSDLQDHDPVKLTPVGLSGDRGSIHQWQLPNYSGVATHNMHLAPSVAPLFPGEQ
ILFFRSTVPGCGGYPNSNIDCLIPQEWVQHFYQEGAPARTDVALLRFINPDTGRVLFECKLHKHGFIT
VAYSGNHDLVMPPNGYFRFESWVNQFHTLAPMGTGSGRRRIQ

GII.21
>KR921942 Hu/GII.21/CUHK-NS-626/HKG/2015
SKTKPFTLPILTIGELTNSRFPAPIDQLYTSPNADVVVQPQNGRCTLDGELQGTTQLLTTAICSYRGT
TSNPTSDYWDDHLLHLVHPNGATYDPTEDVPAPFGTQDFRGILYGVLTQNTQNPRDEVSNSRGIYISS
TSDKFTPKLGTIGLHQVQGDTASNQQSKFTPVGIAVNQNTPFKQWELPNYSGALTLNTNLAPAVGPNF
PGEQILFFRSNVPSVQGNHPTQEIDCLIPQEWVSHFYQESAPSQSDVALVRYVNPDTGRTIFEAKLHR
QGFITIAATGSNPVVVPPNGYFRFDSWVNQFYALAPMGTGNGRRRAQ

GII.23
>KJ19629_Norovirus GII/Hu/JP/2011/GII/Yuzawa/Gira2HS
SKTTPFTLPRLPISEMTNSRFPLVIKGMVVDPNLPLQANFQNGRITLDGELQGTTLPTSTSIGRISGT
HMSSTPSRIIQHEDSGDSTQPRVFNPVWMDLTENNWTEFQPFNDQPAPLGCPDFKAKILGTLIRQPNN
GSYYFDAYLDTRQHGTFAPYTGHAAVHSDQQAGHLAQGYKIQFSPTGIESDQNTDLNQLPDYGGAMTV
SKGLAPAAAPDFPGEMILYFVSDMPVRNPNGERRDTEILCLLPQEMVTHFYEQQAPSQGDVALVRYIN
AETGRVMFEGKLHRNGFFTVSATARTLIVPDGYFRFDSWVNRFYTLSPMGTGNGRRRARMLE

P48

GI.2[P2]
>KF306212.1_Norovirus/Hu/GI.2/Jingzhou/2013401/CHN
MMMASKDVVATNVASNNNANNTSATSRFLSRFKGLGGGASPPSPIKIKSTEMALGLIGRTTPESTGTA
GPPPKQQRDRPPRTQEEVQYGMGWSDRPIDQNVKSWEELDTTVKEEILDNHKEWFDAGGLGPCTMPPT
YERVRDDSPPGEQVKWSARDGVNIGVERLTTVSGPEWNLCPLPPIDLRNMEPASEPTIGDMIEFYEGH
IYHYSIYIGQGKTVGVHSPQAAFSVARVSIQPIAAWWRVCYIPQPKHRLSYDQLKELENEPWPYAAIT
NNCFEFCCQVMNLEDTWLQRRLVTSGRFHHPTQSWSQQTPEFQQDSKLELVRDAILAAVNGLVSQPFK
NFLGKLKPLNVLNILSNCDWTFMGVVEMVILLLELFGVFWNPPDVSNFIASLLPDFHLQ

GI.3[P3]
>KY934262.1_Norovirus_GI_isolate_0304-19
MMMASNDAIAVADVSNNNANTNNDNIGSRLMARIRGRIGPQRGETTTKIKDANMALDLLRRSQTPSPS
RQESPPKSQRDRPPRTASEVKKVLGWDVEPEHQESTAKAWCDLTQEEKEEIMRNNEKLFDAGGITPST
LPSTFERADPVDSPIEQQPVTWSASGGVDIGVNDLTTVRGPFWNMCPLPPLDARNNGPAKEPLIGDMI
EFYEGHIFHYAIYIGQGKTIGVHSPQAAFSIPRITIHPLVAWWRVCYVPTNQQRLTYDQLKELENEPW
PYASITNNCYEFCCRVMALDDTWLERRLVSTGKFNHPTQDWSQDTPDFHQDSKLEMVRDAVLSAINGL
VSQPFKNILSKIKPLNVLNLLSNCDWTFMGVVELIVLLAELFDVFWTPPDISSFIASLLPEFHLQ

GI.4[P4]
>LN854563.1_Norovirus/GI/Hu/NL/2011/GI.4/Groningen
MMMASKDVVAATAASAENTSDNSDSIKNRFFTRLKNLGSNNKPIKIENTHMALNLISRGPSPIPQDKP
PKDQRDKPPRNVAETQQAMGWIDPPMDQNLPTWEELSQTEKQEILKNNSNWFDAGGLAPASIPTGYVK
NTEQQPPDHQVKWSASNGVDLGVGNLTTVPGPAWNLCPMPPIDQRNNGPAKEPLIGDMIEFYEGHIYH
YAIYIGQGKTVGVHSPQAAFSIARITIHPIAAWWRVCYVPTPDQRLNYDQLKELENEPWPYAAVTNNC
YEFCCRILNLQDSWLERRLVTSGRFNHPAQDWSRDTPDFQQDSKLEIVRDAVLAAINGLVSKPFKDLL
NKLKPLNVLNLLSNCDWTFMGVVETIILLMELFGIFWNPPDVSSFIASLLPDMHLQ

GI.6[P11]
>LN854564.1_Norovirus GI/Hu/NL/2012/GI.6/Groningen
MMMASKDVVATNVASNNNANNTSATSRFLSRFRGLGGGASPPNPIKIKSTEMALGLIGKTTQGAAGAN
DLPPKQQRDRPPRTQEEVQYDMGWTERPMDQNVKSWEELDTSTKEEILDSHKEWFDAGGLGPCTMPST
CEQAKDDSPPGEQVKWSVRDGVDLGVNRLTTVSGPEWNLCPLPPIDLRNMEPASEPTIGDMIEFYEGH
IYHYSIYIGQGKTVGVHSPQAAFSVARVTIQPIAAWWRVCYIPQPKHRLSYDQLKELENEPWPYAAIT
NNCFEFCCQVMNLEDTWLQRRLITSGRFHHPSQSWSQQTPEFQQDSKLELVRDAILAAVNGLVSQPFK
NFLGKLKPLNVLNILSNCDWTFMGVVEMVILLLELFGVFWNPPDVSNFVASLLPDFHLQ

GII.2[P2]
>LC209463.1_Norovirus/Hu/GII/JP/2008/GII.P2_GII.2/Hokkaido-15
MKMASNDASAAAAVNSNNDNTKSSSDGMLNNMAVTLKRALGARPKQPAPGEKPPRPPRPPTPELVKRI
PPPPPNDGDEPTVMFSTVGGVSGLPELTTVGQPPEANTAFSVPPLSQRENRDAKEPLTGTILEMWDGE
IYHYGLYVERGLVLGVHKPPAAISIAKVELTPLSLYWRPVYTPQYLIPPDTLRKLHGELFPYTAFENN
CYAFCCWVLDLNDSWLSRRMISRTTGFFRPYQEWNRKPLPTMDDSKLKKVANIVLCALSSLFTRPIKD
IIGKLKPLNILNILASCDWTFAGIVESLILMAELFDVFWTPPDVSAMIAPLLGDYELQ

GII.4[P4]
>KY905331.1_Norovirus/Hu/GII.P4_New_Orleans_2009_GII.4_Sydney_2012/NSW789Z/2016/AU
MKMASNDASAAAVADSNNDTAKSSSDGVLSSMAVTFKRALGARPKQPPPREKPQRPPRPPTPELVKNI
PPSPPNGEDEIVVSYSVKDGVSGLPDLSTVRQPEESNTAFSVPPLNQRENRDAKEPLTGTILEMWDGE
IYHYGLYVERGFVLGVHKPPAAISLAKVELAPLSLYWRPVYTPQYLISPDTLKKLSGETFPYTAFDNN
CYAFCCWVLDLNDSWLSRRMIQRTTGFFRPYQDWNRKPLPTMDDSKIKKVANIFLCALSSLFTRPIKD
IIGKIRPLNILNILASCDWTFAGIVESLILLAELFGVFWTPPDVSAMIAPLLGDYELQ

GII.6[P7]
>LN854568.1_Norovirus/GII/Hu/NL/2014/GII.6/Groningen
MKMASNDATAAFGSQNSVNDSINTAPSNKEEVGAFSNIKVGFKKMLGAVPKGTKAPSSDQHCPLVKIG
TKTLTVPPEPPNGEDTVQFDAKSETVRGLPDLTTVQNEHENTPYTVPPLSEREHRPATEPLPGTILEM
WDGEFYHYSVYVSGGKALGVHKPPAAISLATIELTPISLYWRPVYTPNYLVCPDTLKGLAGEKFPYTA
FSNNCYNFCCWVLELNDTWLSRRSISRTTGFFKPYQSWNRKPLPTVDDGKIKKVANAILCALGSLFSK
PIKDLLGKLKPLNLLHLLASCDWTFAGIVETVILMAELFNIFWTPPDVSSFIASLIGDFELQ

GII.4[P16]
>LC175468.1_Norovirus/Hu/GII/JP/2016/GII.P16_GII.4_Sydney2012/Kawasaki194
MKMASNDATVAVACNNNNDKEKSSGEGLFTNMSSTLKKALGARPKQPAPRDEPQKPPRPPTPELVKRI
PPPPPNGEGEEEPVIRYEVKSGISGLPELTTVPQPDVANTAFSVPPLSLRENREAKEPLTGAILEMWD
GEIYHYGLYVEKGLVLGVHKPPAAISMARVELTPLSLYWRVVYTPQYLISPETLRRLNGEAFPYTAFD
NNCYAFCCWVLDLNDSWLSRRMVQRTTGFFRPYQEWNRKPLPTMDDSKIKKVANIFLCSLSTLFTRPI
KDLIGKIKPLNILNILATCDWTFAGIVESLILFAELFGVFWTPPDVSAMIAPLLGDYELQ

GII.17[P17]
>KT970377.1_Norovirus/GII/isolate/Hu/Guangzhou/GZ2015-L362/CHN/2015
MKMASNDASAAVAGKNNNNDKEKSSSDSLFANMSVTFKKALGARSKQPPPGETKQIQKPPRPPTPELV
KRIPPPPPNGEDEPGIVYKVGEGVSGLPDLTTVVQPDAQNTAYSVPPLSQREVGEAKEPLPGSILEMW

DGEIYHYGLYVERGHVLGVHKPPAAISLAKIEITPLSLYWRVVYTPQYLIDPGTLKNLSGETFPYTAF
DNNCYAFCCWVLDLNDSWLSRRMIQRTTGFFKPYQDWNRKPLPTMDEPKIKKAANAVLCALSSLFTRP
IKDIIGKLRPLNILNILATCDWTFAGIVESLILLAELFGVFWTPPDVSAMIAPLLGDYEMQ

GII.18[P18]
>AY823305.2_Norovirus/swine/GII/OH-QW125/03/US
MMASNDVNVAAGDNTNGNEKQTKMPTSGIFTNVKVGIKKILGDRDCDAQPPAPTPREKPEPPPPPEET
VAGEPPPEGCVAYHKDGDRIDGLPRGPADAEPKCMAYSAPPPHEREDGPAMEPLPGSILEMWDGEIYH
YGLYVNDGLVLGVHRPASAICTATIELTPLSLYWRVVHVPQNVIPSETLKRLAGEKFPYTAFDNNCYA
FCCWVLDLNDSWLSRKMINRTTGFYKPYQVWNRKPLPTMDDGKMKKVTNVLLCALSSLFTRPIKDVIG
SLKPLNLLNLLASCDWTFGGIVETLVLVGELFGIFWTPPDVSAFLAPLLGDYELQ

GII.3[P21]
>LN854569.1_Norovirus/GII/Hu/NL/2014/GII.21/Groningen
MKMASNDASAAAAAKSNNDNAKSSSDGVLSNMAVTFKRALGARPKQPPPSDKPPKPPRPPTPELVKAI
PPPPPNGEDGPIISYNVKGGVSGLPELSTVTQLEENSTAFSVPPLSQRENRDAKEPLTGTILEMWDGE
IYHYGLYVERGLVLGVHKPPAAISLAKIELAPLSLYWRPVYTPQYLISPDTLRKLHGETFPYTAFDNN
CYAFCCWVLDLNDSWLSRRMIQRTTGFFRPYQDWNRKPLPTMDDSKVKKVANVVLCALSSLFTRPIKD
IIGKLKPLNILNILATCDWTFAGIVESLILLAELFGVFWTPPDVSAMIAPLLGDYELQ

GII.4[P31]
>MT232050_Norovirus/Hu/GII/2014/NL
MKMASNDASAAAVANSNNDIAKSSSDGVFSNMAVTFKRALGARPKQPPPKEIPPRPPRPPTPELVKKI
PPPPPNGEDELVVSYSAKDGVSGLPELTTVRQPEETNTAFSVPPLNQRESRDAKEPLTGTIIEMWDGE
IYHYGLYVERGLILGVHKPPAAISLAKVELAPLSLFWRPVYTPQYLISPDTLRRLHGESFPYTAFDNN
CYAFCCWVLDLNDSWLSRRMIQRTTGFFRPYQDWNRKPLPTMDDSKLKKVANIFLCTLSSLFTRPIKD
IIGKLKPLNILNILATCDWTFAGIVESLILLAELFGVFWTPPDVSAMIAPLLGDYELQ

GII.NA2[PNA2]
>MG706448.1_Norovirus/Hu/GII/PNV06929/2008/PER
MKMASNDAATATAGTTSSNSEIHNKDVSNTQNIFANMTVGIKRALGARPKQPPPKPPSTKKEDKTTLP
PIPPPPPNGEDIVITYNKQDDKVSGVPIVSTVETSPLHDTAYSVPPLDQREKVDAKEPLTGSILEMWD
GEIYHYGLYVEKGLVLGVHKPPSALSLARIELTPLSLYWRTVYTPPYLVAPDTLKKLHGESFPYTAFD
NNCYTFCCWVLDLNDSWLNRKCVSRTTGFYRPYQDWNRKPLPTMDESKLKKVANVFLCGLSSLFTRPI
KDLVGRLKPLNILNIVTNCDWTFPGVVEALILLAELFGIFWTPPDISAFVASLLGDYEMQ

GIII.2[P2]
>EU794907.1_Norovirus/Bo/GIII/B309/2003/BEL
MDSSTVKVTGPLAAKASDPDSTFAKLKAKFSALRSPHPPNAQGPSPAETPPTAAPPLGSPPIPLPPGW
EPAEPKREEVDQFPVRFAGGTVHFDVHPPHNAQGPSWNGCPLPPVEQRSTEPPAEPPVGAILEFYEGY
IFHYAIYVGGGKTVGVHSPQMALSVPTIPVQSLSAWWRVAYIPRDPPPRDQLLSLEGERWPYASVTSN
CYTFCCEVLDLDDDWLSRRLVRSGPFHHPSQPWNKAVPEFHQDSKMELVRDAILTALNALVSQPMREL
INVIKPLNVLSILSHCDWTFTGIVEAAVLLAELFGILWQPPDVASFLANLTPECVLQ

GIV.1[P1]
>KC894731.1_Norovirus/Hu/GIV.1/CCDC_GR1113-59/CHN/2011
MKMASNDASVANSNSKTIAANNTTTPKQGGVFANMKIGLKKVLEPKSETPTVRPKDVGKPGGTSPPDD
PPGVTIKYDAQSDTIEGLPNLSTVPQPEARQVKCVPPMAEREVKNAAEPQTGSLLEMYDGSFYHYAIY
IENGLVAGINRPSKALTTATVDVEPIGLWWRVVYTPPFSVSTSALYHLQGEKFPYNAFDNNCYNFCCQ
VLELDDCWMRRKFVQRTTGFFDPYQKWNPKPSQYVADSKLERVGDALLTALGALFSKPIKNIIGKLKP
LNFLNLLSSCDWTFPSIVETIILIAELFDVYWEPPDVTGFLMPLLDDYEFQ

GIV.2[GVI.P1]
>JF781268.1_Norovirus/cat/GIV.2/CU081210E/USA/2010
MASNDAIPAASPKANNEKKSNDGSANNTAYVQPEVGVFANMKVGMQKLLSSGARPKVRPQKPPDRGGT
EQMANAGKPNTARAPVAEGDDHPGLAPQADQGPPPSEVEAVLGSDGAGRVRYIRDQDRIEGLPDLHAA
PFLSEREVKRAPPLDRREFSAAREPPSGSILEMWDGDYYHYAIYLERGLIVGINRPGKALGTATVNIE
PIGLWWRVVHVPRMRVSLPALYKLNGEKFPYNAFNNNCYTFCCQVLELDDSWLNRKYVQRTTGFYDPY
QSWNKKPSIHVADTKMEKVGDMLLTALGALFSRPIKSIINKFKPLNLLNILASCDWTFGGIVEALILI
AELFGVYWEPPDVTAFLMPLLDDFELQ

GVII.1[P1]
>FJ692500.1_Norovirusdog/HKU_Ca026F
MMMASNDVTPCGNEIPKTEINNQSSGRIDRQAETRTTTGTQDGAPPAPAGGFFTRLKDRVKQPKAPPP
QPPGHETQRQEEPVVTYEVRTEAVRGVPDVKAADLPPGYVVPPHLPPIKLRESQDAPEPPTGAILEMW
DGAYYHYGVYVEKGLVLGIHNPPAALSEATVELIPISLFFRVVHVPSNRPSIRALKRLQGEKYPYNAL
THNCYTFCCELLELDDDWEARRLVQRTTGFYDPNQRWNDKPVPLVADSRVVKIADALLAALAGVAAQP
IRDIVNKLKPLNVLHILSSCDWTLSGVVEMVILLAELFGIFWTPPDVTPLIVSLLDDWEMQ

GX.1[P1]
>MF373609.1_Norovirus/bat/isolate_NPIH26
MPPLEDSEDDPFRVSYVRATNTVITFPDMSYCPEDSGAPRPRACPPLFQRAEDPHAAEPQIGAIIEEY
EGQFNHYSLYAGNGEVVGLHSPAAAISMAKVEKRPISLWWRPVYAPKRQYRVAEVVMLVGETVPYVAT
SNNCYDFCVAALGLDDSWLCRKYVTHDTGYYPPKQTWNGNFVDLEQMSKLELVRDAALSAFAAVFSQP
VKDLLNKFKPLNLLHILSSCDWTFSGIVEAVVLVAGLLGVLWSPPDVVAFLAPLMPSLQLQ

P22

GI.2[P2]
>KF306212.1_Norovirus/Hu/GI.2/Jingzhou/2013401/CHN
GKMYDFDDDRVSAFTTMARDNGLGILSMAGLGKKLRGVTTMEGLKNALKGYKISACTIKWQAKVYSLE
SDGNSVNIKEERNILTQQQQSVCAASVALTRLRAARAVAYASCTQSAITSILQIAGSALVVNRAVKRM
FGTRTATLSLEGPPREHKCRVHMAKAAGKGPIGHDDVVEKYGLCETEEDEEVAHTEIPSATME

GI.3[P3]
>KY934262.1_Norovirus_GI_isolate_0304-19
GAVYNFDEDRVSAFTSLARANGLGLLSMATLGKRLRSVKSMEGLRNALVGYKIGECDIIWNTRVYSIK
SDGSTVTIKEKQTPTSPQYQAISTATLALSRLRAARALAYASCLQSAVLSILQVAGSALVVSRAVKRM
FGTRTEQPMLEGKHKEHNCRVHRAEAAGHGPIGHDGVIERYGLCESEQEEEGEQTVELPTANKE

GI.4[P4]
>LN854563.1_Norovirus/GI/Hu/NL/2011/GI.4/Groningen
GQVYDFDADRITAFTSLARANGLGLISMASLGKKLRGVDSVHGLKNALSGYTITPCSIKWQARVYDIE
SDGTNVRIKENTSAQTQRQQSIDTAALALTRLKAARAAAYAACIQSAITTILQMAGSAIVINRAVKRM
FGAHSSTIALEGPPREHRCRAHLAKAAGGGPIGHDDVIDKYGLCETEEEDTSEEINVELPTATSE

GI.6[P11]
>LN854564.1_Norovirus GI/Hu/NL/2012/GI.6/Groningen
GKMYDFDDDRVSAFTTMARDNGLGILSMASLGKKLRGVTSMEGLKNALKGYKIGACTIKWQAKVYSLE
SDGNSVNIREEKNVLTQQQQSVCAASIALTRLRAARAVAYASCIQSAITSILQIAASALVVNRAVKRM
FGTRTAALSLEGPPKEHKCRVHQAKAAGKGPIGHDDVVDKYGLCETEEDEEVVHTEMPSATIEG

GII.2[P2]
>LC209463.1_Norovirus/Hu/GII/JP/2008/GII.P2_GII.2/Hokkaido-15
GPPLSTFNFDRNKIAAFRQLAAENKYGLVDTMRVGNQLRNVTTMDELKKAIKNIIIRKCQIVYSGNTY
TLESDGKGKVVVERVQSTSVQINNEIAGAVHHLRCARVRYYVKCVQEAIYSLLQIAGTVFVTSRIVSR
MNIQNLWSRPLAEDADEATMKEGCPKPREEEEFVVSSDDIKPE

GII.4[P4]
>KY905331.1_Norovirus/Hu/GII.P4_New_Orleans_2009_GII.4_Sydney_2012/NSW789Z/2016/AU
GPALTTFNFDRNKVLAFRQLAAENKYGLMDTMRVGKQLKGVKTMPELKQALKNVSIKKCQIVYSGCTY
MLESDGKGNVKVDRIQSAAVQTNNELAGALHHLRCARIRYYVKCVQEALYSIIQIAGAAFVTTRIVKR
MNIQDLWSKPQVENTEETTSKDGCPKPKDDEEFVISSDDIKTE

GII.6[P7]
>LN854568.1_Norovirus/GII/Hu/NL/2014/GII.6/Groningen
GNDLQTYNFDTNRVSAFRKLAADNKYGIMETMRVGTALKSVKTLEDLKVALRDVKFNECEIIYRNSKY
RVSSNGKGSVSVDKVEDQTSQTANEVGAALLRLRQARARYYVSCFQDLVYTLIQVAGASFVVNRISKR
FCWERWVKPTETQETSESEKEVAQGRWEIEPKDTEPE

GII.4[P16]
>LC175468.1_Norovirus/Hu/GII/JP/2016/GII.P16_GII.4_Sydney2012/Kawasaki194
GPTITTFNFDRNRITAFRQLAAENKYGLVDTMKVGNQLKGVKTMEELKQAIRNVTIKRCRIIYGGSTY
DLESDGKGKVLVEKIKNTSVQTNNELAGALHHLKHARIRYYVKCVQEAVYSIIQIAGAAFVTTRIARR
MNIQELWSKPQLDQNESETKEEAPKSEDDEFIISSKDIKEEG

GII.18[P18]
>AY823305.2_Norovirus/swine/GII/OH-QW125/03/US
GDTGAVYNFDNNKIGAFRKLAADNKYGLMETMRVGAKLQSVKNIDQLRANLRDITIKPCLIIYNSSTY
SLESNGKGNVVIKKVENQVVQSRNTIHTAILRMKQAKIRYYIKCIQEAIYSLLQVAGASFVVARITRK
FTRDAWSHWDNTPDQAPPVPTPPQDASWEIAAADVTPE

GII.3[P21]
>LN854569.1_Norovirus/GII/Hu/NL/2014/GII.21/Groningen
GPTPTTFNFDRNKVLAFRQLAAENKYGLVDTMRVGSQLKNVKTMTELKQALKNISVKKCQLVYGGGTY
TLESDGKGNVHVEKVNNTSVQTNNELSGVLHHLRCARIRYYVKCVQEALYSILQIAGAAFITTRIAKR
TNIQNLWSKPQAEDLEETNNEVGCPKPKNDEEFVVSSDDIKTE

GII.4[P31]
>MT232050_Norovirus/Hu/GII/2014/N
GPALTTFNFDRNKILAFRQLAAENKYGLMDTMRVGKQLKDVKTMSDLKQALKNIAIKKCQIVYNGGTY
TLEADGKGSVKIDKVQSATVQTNNELAGALHHLRCARIRYYVKCVQEALYSIIQIAGAAFVTTRIAKR
MNIQNLWSKPQVEDTEEMANKDGCLKPKDDEEFVVSSDDIKTE

GII.NA2[PNA2]
>MG706448.1_Norovirus/Hu/GII/PNV06929/2008/PER
GPELPIYNFDQNKVASFRKLAADNKYGLMETIKAGNKLKEVKTLDQFKEAVRDIRIKPCRIIYNSTAY
DLESNGSGKVTVKKVGDQKVQANNELYSALNNLRRARIRYYVKCFQEMIYSLLQIAGAAFVTSRMTQR
MNVGKLWTKPERTMKAPTEPPAPPIDDWTINPVDTIPE
